# Usability and feasibility of ADappt: a digital toolkit to support communication on diagnosis and prognosis in memory clinics

**DOI:** 10.1186/s13195-025-01847-y

**Published:** 2025-10-02

**Authors:** Heleen M.A. Hendriksen, Tanja J. de Rijke, Aniek M. van Gils, Marlijn H. de Beer, Femke H. Bouwman, Ana Diaz, Tjeerd Fluitman, Liesbeth Hempenius, Ingrid S. van Maurik, Ruth E. Pel-Littel, Hanneke F.M. Rhodius-Meester, Gerwin Roks, Ellen M.A. Smets, Wiesje M. van der Flier, Leonie N.C. Visser

**Affiliations:** 1 Alzheimer Center Amsterdam, Neurology, Vrije Universiteit Amsterdam, Amsterdam UMC location VUmc, Amsterdam, The Netherlands; 2 Amsterdam Neuroscience, Neurodegeneration, Amsterdam, The Netherlands; 3 Medical Psychology, Amsterdam UMC location University of Amsterdam, Amsterdam, The Netherlands; 4 Quality of Care, Personalized Medicine, Amsterdam Public Health, Amsterdam, The Netherlands; 5 Department of Neurology, Reinier de Graaf Gasthuis, Delft, The Netherlands; 6 Alzheimer Europe, Luxembourg, Luxembourg; 7 Geriatric Center, Medical Center Leeuwarden, Leeuwarden, The Netherlands; 8 Department of Epidemiology and Data Science, Vrije Universiteit Amsterdam, Amsterdam UMC, Amsterdam, The Netherlands; 9 Amsterdam Public Health, Methodology, Amsterdam, The Netherlands; 10 Northwest Academy, Northwest Clinics Alkmaar, Alkmaar, The Netherlands; 11 Vilans Center of Expertise for Long Term Care, Utrecht, The Netherlands; 12 Internal Medicine, Geriatric Medicine Section, Amsterdam Cardiovascular Sciences Institute, Amsterdam UMC location VUmc, Amsterdam, The Netherlands; 13 Department of Geriatric Medicine, The Memory Clinic, Oslo University Hospital, Oslo, Norway; 14 Department of Neurology, ETZ Hospital, Tilburg, The Netherlands; 15 Division of Clinical Geriatrics, Center for Alzheimer Research, Department of Neurobiology, Care Sciences and Society, Karolinska Institutet, Stockholm, Sweden; 16 Department of Bioethics and Health Humanities, Julius Center for Health Sciences and Primary Care, University Medical Center Utrecht, Utrecht University, Utrecht, the Netherlands

**Keywords:** Alzheimer’s disease, Dementia, Mild cognitive impairment, Clinician-patient communication, Diagnosis, Prognosis, Shared decision-making, Biomarkers, Personalized medicine, Usability, Feasibility, Implementation, Digital tool, Memory clinic, Patient and public involvement

## Abstract

**Background:**

ADappt is a digital toolkit for both memory clinic professionals and patients to support communication on diagnosis and prognosis in memory clinics. We aimed to evaluate ADappt’s usability and feasibility in clinical practice.

**Methods:**

In this mixed-methods study, we first assessed usability via think-aloud sessions with ten memory clinic professionals from eight memory clinics, six patients, and one care partner. Think-aloud comments were deductively categorized into: content, navigation, and design. Second, we conducted a feasibility study in four memory clinics. Eight memory clinic professionals recruited 21 patients and 21 care partners. Professionals were instructed to integrate the ADappt-toolkit in their routine. Before their visit, patients received information about the ADappt-patient tools: two video-animations and a question prompt list (QPL). Participants completed questionnaires on usability, satisfaction, and feasibility either after the first consultation (*n* = 14 patients; *n* = 15 care partners), after the disclosure consultation (*n* = 4 patients; *n* = 5 care partners), or after both consultations (*n* = 3 patients; *n* = 1 care partner). Interviews with professionals were conducted and analyzed using thematic content analysis. Third, together with Alzheimer Europe, we co-organized a patient and public involvement (PPI) session with citizens, patients, and care partners to further improve the patient tools.

**Results:**

Professionals found ADappt relevant, easy-to-navigate, and visually appealing. Most think-aloud comments focused on content and navigation, especially regarding the risk calculation tool. Patients indicated the patient tools to be helpful in preparing for consultations. After use in practice, professionals reported acceptable usability (68 ± 14, scale 0-100) and satisfaction (71 ± 10, scale 0-100) with ADappt. Professionals most often used the tool that provides an overview of diagnostic tests with pros and cons (in 15/24(63%) consultations), which they also deemed most helpful (median(IQR): 4(3.75-4), scale 1–5). About half to two-thirds of patients and care partners reported to have received the patient tools (video-animations: *n* = 23/46(50%)); QPL: *n* = 30/46(65%)), of whom a majority used (video-animations: *n* = 16/23(70%)); QPL: *n* = 21/30(70%)) and would recommend them (video-animations: *n* = 15/16(94%); QPL: *n* = 20/21(95%)). The tools helped to express themselves more effectively. The PPI session highlighted the importance of widespread dissemination of the patient tools and through multiple channels.

**Conclusions:**

Our study demonstrates the potential of digital tools to improve medical communication in memory clinics. Taking feedback into account, ADappt is further improved and steps towards implementation are being taken.

**Supplementary Information:**

The online version contains supplementary material available at 10.1186/s13195-025-01847-y.

## Background

Over the past decades, rapid advancements have been made in the diagnosis of dementia, in particular Alzheimer’s disease (AD) [1, 2]. The introduction of new diagnostic tests requires memory clinic professionals to adapt their current practice: larger amounts of information have to be shared, novel decisions need to be made, and more results have to be discussed with patients and care partners. This includes complex, prognostic information. Depending on patients’ information needs, situation, preferences, ability to cope with uncertainty and personal experience with dementia, individual patients have different expectations and goals for the diagnostic trajectory [3]. Effective clinician-patient communication is key for tailoring information and the diagnostic trajectory to the individual patient [4]. This entails a two-way exchange on needs and expectations, the provision of comprehensible information on diagnostic tests with room for shared decision-making, and prognostic communication attuned to the individual [5, 6]. In a recent European survey study, memory clinic professionals reported a need for support in effective clinician-patient communication, such as through skills training or online tools, and many patients and care partners responded favorably regarding the possibility of using (online) communication tools [7, 8]. 

**Fig. 1:**
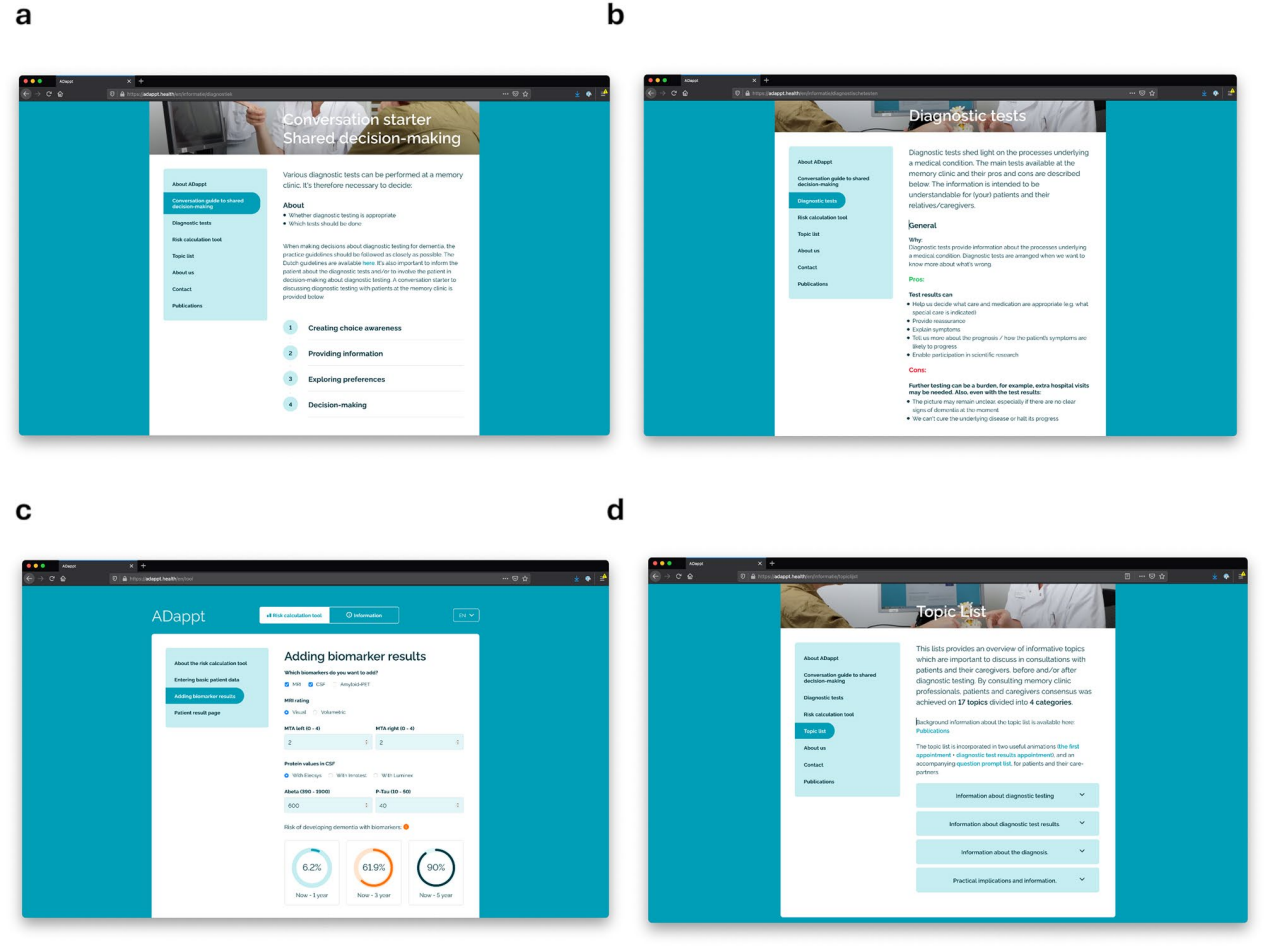
Screenshots of the tools available on ADappt.health [12–14, 17, 18]. From top left to bottom right: **(a)** *Conversation guide to SDM.* This tool outlines the four steps of shared-decision making: (1) creating choice awareness, (2) providing information, (3) exploring preferences, and (4) decision-making. The guide includes example phrases for each step; **(b)** *Diagnostic tests.* This tool provides an description of what can be learned from the available diagnostic tests in the memory clinic. For each test, it explains why it is administered, what it entails, and outlines the pros and cons. The text is written in patient-friendly language, allowing professionals to easily convey this information to patients; **(c)** *Risk calculation tool.* This tool estimates the risk that a patient who presents at the memory clinic with MCI will develop dementia within one, three and five years. The tool includes a patient result page; **(d)** *Topic list*. This tool provides an overview of important topics to discuss during consultations with patients and care partners throughout the diagnostic trajectory. In addition, this tool contains two video-animations (“the first appointment” + “diagnostic test results appointment”) and an accompanying question prompt list to aid patients to prepare for memory clinic consultations. *Note*: All ADappt tools are publicly accessible, except for the risk calculation tool, which requires a log-in. This log-in must be requested from the research team via the ADappt.health website

To provide such support throughout the diagnostic trajectory, we previously developed a web-based toolkit called ‘ADappt’ (www.ADappt.health) [6, 9–11]. ADappt initially comprised tools facilitating memory clinic professionals in shared decision-making and understandable information provision on (pros and cons of) diagnostic test options, and interpretation and communication of diagnostic test results of patients with Mild Cognitive Impairment (MCI) in terms of dementia risk (see Fig. 1). ADappt was developed in an iterative process of co-creation and evaluated positively in a first small pilot study [9]. Since then, lay-out, available languages, and the prediction model underlying the dementia risk tool have been updated. Furthermore, a topic list, containing an overview of topics deemed relevant by both professionals and patients to discuss during consultations, has been added [12]. Based on this topic list, tools for patients and care partners were developed and added, i.e., two video-animations (“the first appointment” + “diagnostic test results appointment”) and an accompanying question prompt list, to aid patients to prepare for memory clinic consultations (see Supplementary Information, Additional file 1) [12]. Additionally, an informative video-animation for patients and care partners on the lumbar puncture procedure was added to the overview of diagnostic tests [13, 14]. 

Building on our iterative development process, the next step involved a three-phase evaluation of ADappt with memory clinic professionals, patients, and care partners. Testing with relevant stakeholders is important to ensure that ADappt meets the needs and expectations of its end-users, aligns with clinical workflows, and addresses potential barriers to adoption [15, 16]. The aim of this study was to evaluate ADappt’s usability and feasibility in clinical practice.

## Methods

### Study design

This study is part of the ABOARD-project (www.aboard-project.nl), which aims to prepare for a future with personalized medicine for AD. ABOARD includes tasks on diagnosis, prediction, prevention, and patient orchestrated care, and this study is part of the latter. We used a mixed-methods design, combining (i) think-aloud sessions to gain in-depth understanding of the thoughts and observations of users whilst interacting with ADappt in a controlled setting [19] and (ii) a feasibility study to obtain insight on the use and facilitators and barriers for implementation in clinical practice [20, 21]. In addition, (iii) we co-organized a patient and public involvement (PPI) session with Alzheimer Europe, during which members of the public, both with and without objective cognitive impairment, provided their thoughts on implementation of the patient tools in clinical practice. The local medical ethics committee of Amsterdam UMC, location VUmc and of the participating hospitals approved the study. The research was conducted in accordance with the Declaration of Helsinki. All research participants provided informed consent.

### Think-aloud sessions

#### Participants

We invited memory clinic professionals to participate via the Dutch memory clinic network. Patients and care partners were recruited at Alzheimer Center Amsterdam, from the Amsterdam Dementia Cohort (ADC) [22]. Patients and care partners had to be aged > 18, proficient in Dutch, and able to provide informed consent.

#### Study procedures

We combined the think-aloud sessions with semi-structured interviews [19]. Participants were asked to use ADappt by means of a fictional patient case (see Supplementary Information, Additional file 2, Textbox 1 and 2). For professionals, the task involved preparing the fictional patient’s first consultation and the disclosure consultation, engaging with the tools that they deemed relevant (see Supplementary Information, Additional file 2, Textbox 1 and Additional file 3). For patients and care partners, the task included preparing for the first and the disclosure consultation, while imagining themselves as the patient in the fictional case. They were instructed to watch the animations, use the question prompt list, and provide comments on the fictional patient’s result page (see Supplementary Information, Additional file 2, Textbox 2). All participants were asked to verbalize their thoughts and observations while using ADappt. Following the think-aloud session, a semi-structured interview was conducted with each participant to reflect and elaborate on their experiences, and to gather additional information based on the researcher’s observations (see Supplementary Information, Additional file 2, Textbox 3) [23, 24]. Sessions lasted approximately 30 to 60 min. Due to COVID restrictions at the time, all but one of the sessions took place via video-conferencing. When applicable, patients and care partners were reimbursed for travel expenses. Sessions were conducted either one-on-one (HH (psychologist) with professional/patient) or one-on-two (HH with patient + care partner).

### Feasibility study in memory clinic practice

#### Participants

We conducted a multicenter study in four Dutch memory clinics (Reinier de Graaf Ziekenhuis, Elisabeth Tweesteden Ziekenhuis, Medisch Centrum Leeuwarden, and Amsterdam University Medical Center (UMC)). Professionals were included if they were involved in the diagnostic work-up of dementia. Professionals were instructed to invite all patients who were scheduled for an appointment during a specified timeframe for participation (4 weeks to 8 weeks, depending on the set-up and the number of new patients seen at the clinic). The appointment could be the first consultation at the clinic or the test result disclosure consultation, or both. At their scheduled visit, patients and their accompanying care partners were invited by the professional for to participate in the study and provide informed consent. Patients and care partners had to be aged > 18, proficient in Dutch, and able to provide informed consent.

#### Study procedures

Memory clinic professionals were asked to integrate ADappt in their daily routine, when preparing for, or during either first or disclosure consultations. The diagnostic work-up was performed according to local practice. HH and TR provided an on-site training on how to use ADappt. For this, we developed a practice guide in the form of a clickable PDF comprising a: (1) factsheet, (2) workflow, and (3) slide-deck (see Supplementary Information, Additional files 4–7). Patients and their care partners were invited for the study by their memory clinic prior to their visit, by means of an information letter and an additional brochure, which included detailed instructions on how to access the video-animations and the question prompt list online (see Supplementary Information, Additional file 8). Additionally, a printed version of the question prompt list was provided (see Supplementary Information, Additional file 1).

#### Measures

Memory clinic professionals completed a short questionnaire (satisfaction, feasibility) after each consultation with a participating patient, and a more detailed questionnaire at both the beginning (sociodemographics, professional characteristics, memory clinic characteristics) and the end of the study period (usability). Usability was assessed using the System Usability Scale (SUS), a 10-item standardized questionnaire with 5-point Likert scale responses (1 = Strongly disagree, 5 = Strongly agree) [25]. In addition, professionals rated the usefulness of each ADappt tool, and indicated whether they would like to use the tool in daily practice, whether they could easily integrate it, and whether they encountered any issues in using ADappt, such as technical problems. Participating patients and care partners received a questionnaire (usability, satisfaction, feasibility) after their consultation, which they could return by post or digitally. Satisfaction was assessed using the Patient Satisfaction Questionnaire (PSQ – Dutch version; 5 items, 0 = Not at all, 100 = Very much) [26]. In addition, participants reported their opinions on ADappt using study-specific items. These assessed their awareness and use of the patient tools, the extent to which the tools helped them prepare for the consultation (4-point Likert scale, 1 = Not at all, 2 = A little, 3 = Quite a bit, 4 = Very much), and whether they would recommend the tools to other patients (yes/no, with open-ended explanation). After the study period, we conducted semi-structured interviews via phone or video conferencing with professionals about their experiences and ADappt’s usability and feasibility. For a detailed description of the questionnaires and interview guide, see Supplementary Information, Additional file 2, Table 1 and Textbox 4.

### PPI session

#### Participants

Together with Alzheimer Europe, we co-organized an international patient and public involvement (PPI) session with 18 members of the Advisory Boards of the EU-Fingers (www.eufingers.com), LETHE (www.lethe-project.eu), and Multi-MeMo (www.multi-memo.eu) consortium projects to identify ways to foster implementation of the patient tools (video-animations and question prompt list) in clinical practice. The Advisory Boards include patients, care partners, and individuals interested in brain health from nine different European countries.

#### Study procedures

We held discussions at four small tables, each with 4–5 members and two facilitators (one main facilitator and one co-facilitator), focusing on when, from whom (e.g., general practitioners or memory clinics), and how (e.g., digital or postal) patients would prefer to receive these tools. Participants watched the video-animations together and were offered a printed question prompt list. The main facilitators (HH, LV, TR, AD) wrote down opinions, and HH summarized the results.

### Data analysis

**Fig. 2:**
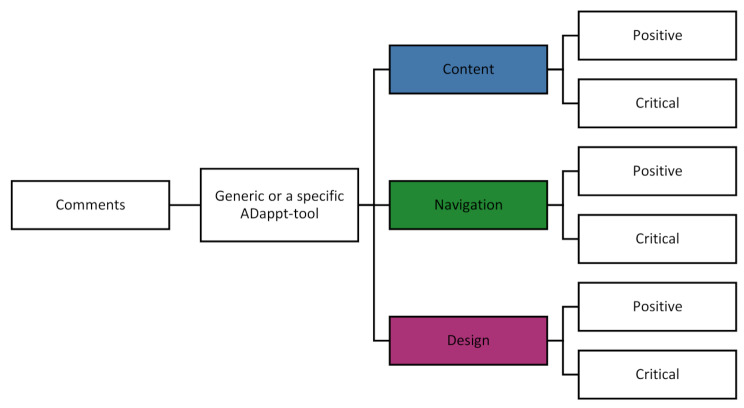
Deductive code tree

Think-aloud sessions were transcribed and deductively analyzed according to the code tree in Fig. 2, using MAXQDA2020 [27, 28]. We determined whether a comment was (1) generic or related to a specific ADappt-tool, (2) related to content (comments related to information content, e.g., problems with understanding the information, incomplete information, or inaccurate information), navigation (comments related to finding the required information on the website, e.g., a poorly organized home page, unclear link labels, and problems with the structure), or design (comments related to the functionality of the design, e.g., an illegible font, or an unclear lay out), and (3) positive or critical [27, 28]. The interviews of the feasibility study and the open-ended questions from the questionnaires were jointly analyzed with thematic content analysis [29]. From participants’ critical feedback, we derived suggestions for improvement. Coding was done independently and hereafter discussed until consensus was reached by HH (psychologist), AG (medical doctor), and TF (health scientist), all trained in qualitative analysis. We used descriptive statistics to report characteristics and survey responses using SPSS-statistics software version 28.

## Results

Professionals, patients, and care partners’ characteristics are shown in Table 1. Ten professionals from eight memory clinics (11 ± 6yrs experience), six patients, and one care partner participated in the think-aloud sessions. Patients were on average 68 ± 8yrs years old and 33% was female. Eight professionals (12 ± 7yrs experience), 21 patients and 21 care partners participated in the feasibility study. Patients and care partners were on average 69 ± 8 and 60 ± 15 years old, respectively. Most patients were male (81%). There was no overlap in professionals participating in the think-aloud sessions and in the feasibility study.

**Table 1:** Sample characteristics for think-aloud sessions and feasibility study

	Think-aloud	Feasibility study	
**Professionals**	***N*** = **10**	***N*** = **8**	
Age	44 ± 6	43 ± 11	
Female, n (%)	10 (100%)	7 (88%)	
Years of clinical experience	11 ± 6	12 ± 7	
Profession, n (%)			
Medical specialist	9 (90%)	4 (50%)	
Physician, without or currently in specialist training	-	2 (25%)	
Specialized nurse	1 (10%)	2 (25%)	
Medical specialty, n (%)			
Neurology	3 (30%)	5 (63%)	
Internal/Geriatric medicine	7 (70%)	3 (38%)	
**Patients and care partners**	***N*** = **6 patients**	***N*** = **21 patients**	***N*** = **21 care partners**
Age	68 ± 8yrs	69 ± 8yrs	60 ± 15yrs
Female, n (%)	2 (33%)	4 (19%)	17 (81%)
Education, median (IQR)	6 (6–6)	5 (5–6)	5 (5–6)
Information need (scale 1–10)	N/a	8.4 ± 1.4	8.9 ± 1.2
*Type of consultation*	N/a		
First consultation		14 (67%)	15 (71%)
Disclosure consultation		4 (19%)	5 (24%)
Both		3 (14%)	1 (5%)
Diagnosis	5 SCD, 1 AD dementia	2 SCD, 4 MCI, 1 AD dementia, 14 unknown	

Data are presented as mean ± SD, n (%), or median (IQR). Education is rated using the Dutch Verhage system, ranging from 1-7 [30]. *Abbreviations*: SCD: Subjective Cognitive Decline, MCI: Mild Cognitive Impairment, AD: Alzheimer’s disease

In the next sections, we first focus on the professionals perspectives as derived from the think-aloud and feasibility study, and subsequently on the patient and care partner perspectives.

### Professionals

#### Think-aloud sessions

Regarding the *content*, professionals were generally satisfied with the information provided through ADappt and recognized its potential benefit for memory clinic practice. They thought ADappt could serve well as a training or a reference for junior doctors with less experience with regards to communication and interpretation of test results. Suggestions for improvement with regard to the content were mostly related to the dementia risk calculation tool: Some professionals indicated to use the Montreal Cognitive Assessment (MoCA), whereas the tool assumes that the Mini-Mental State Examination (MMSE) is available. In addition, some professionals were not aware of the cut-offs for certain biomarkers, e.g., amyloid; they would like the tool to provide this information. With regard to *navigation*, professionals considered ADappt to be easily accessible with an intuitive flow from one page to another. Suggestions for improvement regarding navigation mostly concerned the dementia risk calculation tool: professionals were disappointed that data entered in this tool was not saved after leaving the page. In addition, not all professionals succeeded in entering the amyloid-PET result of the hypothetical patient case in the tool. Regarding *design*, professionals described ADappt as an easy-to-read toolbox with appealing visuals. No suggestions for improvement were made. Positive and critical comments on specific ADappt-tools can be found in Table 2.

#### Feasibility study in memory clinic practice

Professionals reported to use ADappt in 16 out of 24 (67%) consultations. The most frequently used tool was the overview of diagnostic tests with pros and cons (15/24, 63%), which was also rated as the most helpful (median(IQR): 4(3.75-4), scale 1–5). Other tools were considered useful but were used less frequently, including the conversation guide to shared decision-making (9/24, 38%; 4(3–4)), the topic list (5/24, 21%; 4(3–4)), and the risk calculation tool, which is only applicable in patients with a diagnosis of MCI (1/24, 4%; 4(3–4)). Overall, reported satisfaction with ADappt after each consultation was high, with a mean score of 71 ± 10 (range 50–85, scale 0–100).

Professionals reported usability ranging from OK to good for ADappt (68 ± 14, range 50–83, System Usability Scale [31, 32], scale 0-100). They were more positive than negative about wanting to use ADappt in daily practice (median(IQR): 3.5(3–4), scale 1–5) and about how easily ADappt could be integrated in their routine (3.5(2.75-4), scale 1–5). Technical issues were rarely reported (1(1-1.25)), as were problems with interpreting results (1(1-1.25)), communication with patient (1(1-2.25)) or with the use of ADappt (1.5(1–2)).

Interviews provided further insights into the barriers and facilitators for using ADappt and its tools. Identified barriers include first, the limited time available during consultations with too many tasks to fit in. Second, establishing a routine and becoming familiar with ADappt was considered challenging when a low number of patients was seen per week. Third, professionals deemed not all patients suitable for the tool: certain patient characteristics, such as low digital literacy, emotional burden, or difficulty making decisions, posed challenges. Low digital literacy was often attributed to older patients, leading professionals to feel that ADappt was more suitable for younger patients.

- *“Sometimes it can also be challenging for the patient*,* because making choices is difficult. So that makes it a bit tricky when you want to engage in shared decision-making. Some patients find it very difficult*,* while others really want to have that choice.” (interview feasibility study*,* clinician 4)*
- *“That was a younger person*,* and they also had a son with them who could just understand it all a bit better*,* so to speak. I think something like that isn’t really suitable for an 85-year-old with little interest in ICT*,* to put it bluntly. But certainly for the somewhat younger people*,* right? People come to us at a relatively young age*,* so it can definitely be of added value*,* yes”* (*interview feasibility study*,* clinician 5*)

Professionals identified several facilitators for implementing ADappt. Regularly seeing patients for whom ADappt was deemed relevant helped them to integrate the tool into their daily routine, as did receiving positive feedback from patients during consultations, which in turn encouraged using the tools more often. They suggested that linking ADappt with the Electronic Health Record (EHR) would facilitate its integration into their workflow. Additionally, they recommended on-the-job training and broader implementation across the entire memory clinic department, rather than involving only a few professionals, so that positive reinforcements to use ADappt would occur more frequently. Lastly, professionals suggested creating a general instructional video for professionals about the intended use of ADappt and its tools.

- *“Well*,* you can*,* of course*,* imagine that these kinds of things could simply be integrated into the EHR*,* right? The computer system. So that you don’t have to think every time about filling something in*,* but if you could*,* for example*,* create a link to the ADappt tool*,* you could open it directly without first having to think*,* ‘Oh*,* what was the web address again?’ It would then work automatically*,* so to speak.” (interview feasibility study*,* clinician 3)*.
- *“It took me a while to figure it out*,* to get a sense of how it… what I could use and where to find it*,* so to speak. And now that I know*,* it actually seems quite logical. You think*,* why couldn’t I find it? So what exactly could be improved there*,* or maybe… perhaps having some kind of instructional video or something*,* right?” (interview feasibility study*,* clinician 2)*.

### Patients and care partners

#### Think-aloud sessions

Patients and care partners found the video-animations and question prompt list helpful in preparing memory clinic visits. They highlighted the importance of having materials available at home, allowing them to re-read or re-watch content, especially given the nature of the cognitive complaints that lead patients to a memory clinic. Participants unanimously indicated they would like to see these tools implemented in routine practice. When critical feedback was provided on the video-animations and question prompt list, this mainly reflected a wish for including more detailed information and additional topics. In contrast, comments on the Result page were more substantial, addressing issues such as the complexity of the language, or a preference for a different visualization of the test results (see Table 3). Participants provided suggestions for improving each tool, while acknowledging that each patient is different and might have their own preferences.

#### Feasibility study in memory clinic practice

In the multicenter study, patients and care partners were highly satisfied with the consultations in general (81 ± 14, range 50-100, Patient Satisfaction Questionnaire, scale 0‐100^26^). Many patients noted that the tools helped them express themselves more effectively during the consultations:

- *“If you know what to expect*,* it makes you less insecure + invites you to formulate questions” (female*,* 57 years*,* Subjective Cognitive Decline)*.
- *“You’re usually a bit tense during such an appointment*,* which makes you quickly forget to ask something you’d like to know.” (male*,* 59 years*,* care partner)*.

Regarding the first consultation, more than half (19/33; 58%) of patients and care partners reported to be aware of the video-animation. Among those, the majority (14/19; 74%) had watched it and felt it helped them to some extent to prepare for the appointment (2.5(2–3), median(IQR), scale 1–4) and almost all (13/14; 93%) would recommend the video to others. Regarding the question prompt list, the majority (25/33; 76%) of patients and care partners reported to be aware of this tool. Of those, many (17/25; 68%) had used it, reporting that it helped them somewhat in preparing for the appointment (2.0(2–3)), with nearly all (16/17; 94%) recommending it to others.

For the disclosure consultation, four (4/13; 31%) patients and care partners reported being aware of the video-animation, and half of them (2/4; 50%) had used it, finding it helpful in preparing for the appointment (3(3–3)). Five (5/13; 38%) patients and care partners reported to be aware of the question prompt list, with four (4/5; 80%) using it and finding it useful to prepare for the appointment (2.5(2–3)). All indicated they would recommend both the video-animation and the question prompt list to others.

#### PPI session

Finally, we evaluated the tools in an international panel of patients, care partners, and individuals interested in brain health. Regarding the timing (“when”), participants recommended providing the video-animations and question prompt list well before the first memory clinic appointment, e.g., at the point of referral by the general practitioner (GP) or medical specialist, or even before visiting the GP. Several participants emphasized their preference for receiving information more than once: initially when the appointment is made and again just before the visit. Participants suggested sending the second video-animation on what to expect during the results consultation when scheduling this appointment and again shortly before the actual appointment.

Concerning the source (“who”), participants favored collaboration between public health authorities, including GPs, memory clinics, and community nurses. Of note, the national organization of dementia diagnostics and care varies between countries. Many stressed the importance of a trusted source as a counterbalance to potentially misleading information that can be found online. In addition, they proposed airing the video-animations in public spaces (e.g., buses, trains, TV ads) to reduce stigma and encourage people to seek referrals if they have concerns.

In terms of distribution (“how”), preferred methods included sending materials by post (with QR codes for the online videos), by email, and/or by making them publicly available, e.g., on a website. Additionally, participants suggested showing the videos in waiting rooms to reinforce expectations and key questions patients should ask during appointments.

**Table 2:** Feedback from professionals regarding the content, navigation, or design of specific ADappt-tools

ADappt tool	Positive feedback	Critical feedback	Suggestions for improvement	Issue type
**Conversation guide to SDM**	Raises awareness of shared-decision making; relevant to consultation	Confusing name (overlap with QPL); less useful with standardized diagnostic protocols; challenging to incorporate (requires behavioral change)	Rename tool; consider additional training (e.g., e-learning, skills training)	Content
**Diagnostic tests**	Easy to apply: practical phrasing; useful, especially for tests less often discussed (e.g., LP)	--	--	--
**Risk calculation tool**	Helpful in interpretating results and whom to follow-up; increases confidence when discussing prognosis	Lack of possibility to enter MOCA; generalizability concerns; cut-off values for biomarkers missing; data storage: data was not saved after leaving the page; unclear which biomarker (e.g., MRI, CSF, amyloid-PET) combinations are possible; log-in issues	Add MOCA option; add biomarker cut-off values; enable interim data storage; show possible biomarker combinations clearly	Content, Navigation, Design
**Topic list**	Helpful memory aid during preparation and consultation	Title did not reflect content well; tool often used via QPL instead	Rename tool (e.g., “Checklist”)	Content
**Patient tools** (QPL, video-animations, result page)	Informative, clear, patient-friendly; risk visualizations appreciated; QPL helps structure consultation; patients better prepared	Concern about inducing worry; no insight into video-animation use; QPL not digital; patients ticked boxes of questions that were not addressable yet during the first consultation	Add QR-codes of video-animations to QPL; enable digital QPL; incorporate separate headings in QPL	Content, Navigation, Design

Abbreviations: SDM: shared-decision making, LP: lumbar puncture, MOCA: Montreal Cognitive Assessment, MRI: Magnetic Resonance Imaging, CSF: cerebrospinal fluid, PET: positron emission tomography, QPL: question prompt list

**Table 3:** Feedback from patients and care partners regarding the content, navigation, or design of the patient tools

ADappt tool	Positive feedback	Critical feedback	Suggestions for improvement	Issue type
**Question prompt list**	Helpful memory aid; puts your own questions in a larger perspective; appreciation for open-ended question field; complete and clear; supports preparation and expectations	Missing example questions for post-diagnosis phase; some wording unclear; wish for digital completion	Develop post-diagnostic QPL version; revise unclear wording; enable digital submission	Content, Navigation
**Video-animations**	Clear and concise; helpful visual and auditory explanation (in addition to just reading); not too long	Wish for more information (e.g., the different diagnostic tests and their duration, post-diagnostic support); confusion due to YouTube autoplay	Develop video(s) on diagnostic tests and post-diagnostic support; prevent or warn for autoplay of unrelated YouTube videos	Content, Navigation
**Result page**	Provides clarity; supports future planning; appreciated risk communication; helpful to re-read at home; visuals clear	Complex or redundant language; some information lacks personalization (e.g., own MRI); LP cut-off values lacking; wish for more outcomes than dementia risk (e.g., driving); follow-up information lacking; layout issues (e.g., unclear test result highlights, blurry MRI image)	Simplify language; personalize where possible; improve MRI image; adjust test result presentation	Content, Design

Abbreviations: LP: lumbar puncture, MRI: Magnetic Resonance Imaging, QPL: question prompt list

## Discussion

In this mixed-methods study, we demonstrated that ADappt, an online toolkit to support communication on diagnosis and prognosis in the memory clinic, meets communication support needs of both professionals and patients and care partners, is easy to navigate, and visually appealing. Professionals deemed the diagnostic tests tool most valuable, providing easy-to-understand information on diagnostic tests along with their pros and cons. Patients and care partners positively evaluated the video-animations and the question prompt list, considering them helpful in better preparing for the visit and enabling them to express themselves more effectively. In addition, we found that the tools within ADappt are at varying stages of usability and feasibility, requiring tailored approaches for further improvement and implementation.

### Tools facilitating the discussion of important topics during the consultation

The development of patient and care partner directed tools to facilitate discussions on key topics was motivated by findings that patients and care partners ask few questions during consultations, yet often leave with unmet information needs [10, 12, 33]. Throughout this three-phase study, patients and care partners consistently valued the question prompt list and video-animations as supportive tools to express themselves more effectively during a consultation. In addition, there was alignment between professionals, patients, and care partners regarding several aspects of the patient tools. Both groups appreciated the clarity and supportive nature of the materials, particularly the visualizations and the role of the QPL in preparing and structuring the consultation. Similar findings have been reported in other medical specialties, where the use of question prompt lists and animated videos was associated with improved knowledge, increased confidence in asking questions, higher satisfaction, and reduced anxiety [34, 35]. This demonstrates that such tools are broadly supported across healthcare settings.

Several lessons emerged from this study to enhance the feasibility of these patient tools. A substantial number of potential users were unaware of the tools, particularly the video-animations. While the question prompt list was included with the appointment letter, accessing the video-animations required following instructions in a separate brochure. To improve accessibility, QR codes linking directly to the video-animations have now been added to the question prompt list. We also developed the topic list tool as a reminder for professionals of which topics are considered important by patients and care partners, but it appeared redundant when the question prompt list is already in use. Participants in the PPI session emphasized their need for close collaboration among healthcare providers and widespread dissemination of these tools to raise awareness. They suggested making the tools available not only in memory clinics, but also through general practitioners and even before a formal diagnosis – thus ensuring timely access to relevant information for the general public, with the potential added benefit of reducing stigma. Currently, limited interaction between memory clinics, other medical specialists, and general practitioners hinders integration. A shared digital platform could foster collaboration, enabling patients and professionals to access the same resources and supporting patients in taking a more proactive role in managing their healthcare [4, 36]. The wish for digital accessibility of the patient tools (e.g., completing the QPL online) was mentioned by both professionals, patients, and care partners in our study. To support effective implementation, we have established an “Implementation Studio” within the Dutch Memory Clinic Network. This initiative provides support in the further improvement and implementation of the ADappt patient tools, and fosters interaction between memory clinics.

### Tools to clearly explain diagnostic procedures and facilitate shared-decision making

Memory clinic professionals frequently discuss diagnostic possibilities with patients, and this will likely increase both in number and in complexity in the future when disease-modifying treatments become available [1, 2]. The diagnostic tests tool is easily applicable, which likely contributed to its frequent use and positive evaluation [10]. It is important that this tool remains up-to-date and incorporates new tests, such as blood-based biomarkers [37]. Digital tools, like ADappt, then have the potential to train professionals more quickly and reduce the time between innovation and implementation.

Informing the patient, for example about diagnostic test options, is one of the four steps of shared decision-making (SDM). Shared decision-making is a collaborative process where professionals and patients make decisions together based on available evidence, and is advised in situations where the pros and cons of medical decisions are closely balanced [6]. The conversation guide to SDM tool raised professionals’ awareness of the importance of SDM. Professionals indicated a need for additional support, such as an e-learning or skills training, to facilitate incorporating SDM and the tool in their consultations. This finding aligns with existing literature, which indicates that while professionals generally prefer active patient involvement, they often find it challenging to engage them effectively [10, 38, 39]. Memory clinic professionals are open to use tools or participate in training to improve their SDM skills, and in oncology, such tools and training have already shown to enhance the implementation of SDM [7, 40]. Currently, the conversation guide to SDM is a relatively static tool that provides dropdown menus for each step of the SDM process, offering short descriptions and example phrases. Future work should co-design a more dynamic format or training that better supports memory clinic professionals in implementing SDM behavior in practice.

### Dementia risk calculation tool and patient result page

The dementia risk calculation tool received the most feedback during the think-aloud sessions and it was used least frequently by professionals in the feasibility study. Three key factors may explain this: (1) the tool was only applicable to MCI patients, who represent a minority of memory clinic patients; (2) the tool requires extensive user interaction, which necessitates more practice; and (3) if there is still significant critical feedback on its usability, it is less likely that it is used in practice [1, 41]. The patient result page was positively received as a clear and welcoming way to explain prognosis but could benefit from improved design and more accessible language. The appreciation of a patient result page by both professionals and patients and care partners was also found in a recent usability study on a clinical decision support system, further highlighting the widespread need for patient tools [42]. 

To address the first factor, we recently developed a prognostic model for patients with MCI or mild dementia due to AD, making the group of patients where such a tool could be of use larger [43]. This model can be used to discuss (the uncertainty of) prognosis as well as how a putative treatment that would alter the course by ~ 30% would impact this. As supported by literature and confirmed in our think-aloud sessions, many patients express a need for prognostic information, regardless of disease stage [10, 33, 44–46]. This prediction model has now also been integrated in the latest version of ADappt, along with patient-friendly information and a video-animation about newly emerging anti-amyloid treatment. To address the second and third factors, we explore the addition of crosswalk tables to convert MoCA scores to MMSE scores [47]. In addition, we could provide more guidance to increase confidence in the use of the tool by providing cut-offs for specific biomarkers and data on the reliability of the prediction models, a need also identified in a recent usability study on a clinical decision support system [42]. 

### Strengths and limitations

This study has several limitations. First, there is potential selection bias in the think-aloud sessions, as participants with an interest in communication tools or with relatively minor cognitive deficits may have been more likely to participate. Second, for many participants, their final diagnosis remained unknown, which could limit the generalizability of our findings. Finally, the small sample size in the feasibility study reduces the robustness of results. In particular the international nature of the interactive PPI panel session contributes to the generalizability of results. Despite these limitations, the study has notable strengths. The mixed-methods design, combining qualitative and quantitative approaches, provided complementary insights into the usability and feasibility of the tools in ADappt. Another strength is the iterative development process, which consistently involved key stakeholders, including memory clinic professionals, patients, and care partners [9, 15]. Finally, the diversity of the professionals’ sample, which included participants from various medical specialties (neurology and internal/geriatric medicine) and with differing levels of clinical experience, enriched the study’s findings.

### Future directions

We will continue our efforts with the Implementation Studio within the Dutch Memory Clinic Network to provide support in the implementation of the ADappt patient tools and stimulate the exchange of best practices regarding patient information across memory clinics. Additionally, we will focus on keeping the diagnostic tests tool up to date, explore revising the conversation guide to SDM, and enhancing the usability of the risk calculation tool and its associated patient result page. At the European level, advancements will be made within the IHI-PROMINENT and AD-RIDDLE studies, focusing on advancing the diagnosis, treatment, and management of neurodegenerative diseases by developing digital platforms and evidence-based tools that integrate diverse data sources, enable personalized medicine, and promote collaboration among healthcare professionals, patients, and care partners [48, 49]. With these ongoing initiatives, we will continue working on the refinement and development of ADappt, ultimately aiming to enhance the diagnostic trajectory in memory clinics for professionals, patients, and their care partners.

## Conclusions

In conclusion, our study provides valuable insights into the usability and feasibility of ADappt in memory clinic practice. The findings indicated that the ADappt addresses a need, is easy to navigate, and visually appealing. The different tools available come with different barriers and facilitators, calling for tailored (implementation) strategies. The diagnostic trajectory of Alzheimer’s disease and dementia can be uncertain and complex. Throughout this journey easily accessible, reliable, and up to date information is crucial. Most importantly, we show support for the notion that online tools can help to provide reliable, tailored, and accessible information to memory clinic patients and enhance clinician-patient communication in the clinic.

## Supplementary Information

Below is the link to the electronic supplementary material.

Supplementary Material 1

Supplementary Material 2

Supplementary Material 3

Supplementary Material 4

Supplementary Material 5

Supplementary Material 6

Supplementary Material 7

Supplementary Material 8

## Data Availability

The data acquired during the current study is available from the corresponding author on reasonable request.

## Abbreviations

ADC: Amsterdam Dementia Cohort
AD: Alzheimer’s disease
CSF: Cerebrospinal fluid
EHR: Electronic Health Record
GP: General practitioner
LP: Lumbar puncture
MCI: Mild Cognitive Impairment
MMSE: Mini-Mental State Examination
MoCA: Montreal Cognitive Assessment
MRI: Magnetic Resonance Imaging
PET: Positron Emission Tomography
PPI: Patient and public involvement
QPL: Question prompt list
SCD: Subjective Cognitive Decline
SDM: Shared-decision making
