# Supplementary material for: Usability and feasibility of ADappt: a digital toolkit to support communication on diagnosis and prognosis in memory clinics
Source: Alzheimers Res Ther. 2025 Oct 2;17:218. doi: 10.1186/s13195-025-01847-y (PMC12492680; doi:10.1186/s13195-025-01847-y)
Supplement: Supplementary file 1 — Supplementary Material 1 [file 13195_2025_1847_MOESM1_ESM.pdf]

Supplementary Information, Additional file 2

Textbox 1. Description of the task for professionals

The initial consultation

You receive a referral from a general practitioner for a 56-year-old woman experiencing increasing forgetfulness over the past year. The GP administered the MMSE, on which she scored 28 points. The patient works as a social pedagogical worker (SPW) at a daycare center and experiences a high workload. She is concerned that she may have Alzheimer's disease, a fear reinforced by her personal experience with her mother, who suffered from the condition.

Your task is to use ADappt to prepare for the initial consultation as you would for this patient. You are encouraged to continuously verbalize your thoughts and indicate when you have completed the task.

The follow-up consultation (disclosure of results)

Based on the initial consultation, your differential diagnosis includes subjective cognitive decline (SCD) or mild cognitive impairment (MCI) due to Alzheimer's disease, or potentially mood-related issues. The cognitive test results are as follows: MMSE: 27/30, MoCA: 25/30, GDS: 7/15, FAB: 17/18, and Naming test: 19/20.

Together with the patient, you have decided to request a neuropsychological assessment (NPA), an MRI scan, and a lumbar puncture. Additionally, an amyloid-PET scan was performed as part of a research study.

The test results are as follows:

NPA: Impairments are most pronounced in learning ability. Additionally, visuoconstructive abilities appear below expectation. Performance on attention, concentration, processing speed, language, executive functions, and visuospatial functions is within the normal range.

Brain MRI: Hippocampal atrophy left/right grade 1/1, global atrophy grade 1, parietal atrophy grade 2/2, vascular white matter changes (Fazekas) grade 1.

Lumbar puncture: Abeta 1-42 (Elecsys): 700, Total Tau: 251, pTau-181: 24

Amyloid-PET: Positive

Your task is to use ADappt to prepare for this follow-up consultation as you would in clinical practice. You are encouraged to continuously verbalize your thoughts and indicate when you have completed the task.

Textbox 2. Description of the task for patients and care partners

The first appointment
A 56-year-old woman is referred to the memory clinic by her general practitioner. She is experiencing short-term memory problems and is concerned that she may have Alzheimer's disease, just like her mother. Her concerns are reinforced by the fact that she has witnessed the disease up close in her mother.

You may put yourself in the situation of this woman. You are asked to use the animation videos and the question prompt list to prepare for the first appointment as you would in this woman's case. First, I will show you the animation video designed to help prepare for the first appointment. You may pause the video as often as you like to provide comments.

Show animation video 'The first appointment'

Now, I will give you the question prompt list, which you may use to prepare for the first appointment. You may keep talking continuously and indicate yourself when you are ready.

The results appointment
Following the first appointment, several diagnostic tests are conducted: a neuropsychological assessment, an MRI scan, and a lumbar puncture. Additionally, an amyloid-PET scan is performed as part of a research study.

Once again, I ask you to put yourself in the situation of this woman. You are asked to use the animation videos and the question prompt list to prepare for the results appointment as you would in this woman's case. First, I will show you the animation video designed to help prepare for the results appointment. You may pause the video as often as you like to provide comments.

Show animation video 'The results appointment'

Now, I will give you the question prompt list, which you may use to prepare for the results appointment. You may keep talking continuously and indicate yourself when you are ready.

Hand over question prompt list

This woman ultimately receives the diagnosis of mild cognitive impairment due to Alzheimer's disease. The doctor has printed a results page with an explanation of each test result and an overall summary. You will now see this results page and may say out loud what you think about it. You may indicate yourself when you are ready.

Hand over patient result page

Textbox 3. Script semi-structured interviews following the think-aloud sessions with professionals, patients, and care partners.

Professionals
1. Which modules from ADappt did you find useful? Which not?
2. Were you annoyed by something while using ADappt?
3. What do you think are positive and negative aspects of ADappt?
4. Do you have any recommendations on how to improve ADappt?
5. Would you like and be able to use the current version of ADappt in clinical practice? Why (not)?

Patients and care partners
1. Did you find the animations, the question prompt list and the result page understandable?
2. What do you think are positive and negative aspects of the animations, the question prompt list and the result page?
3. Were you annoyed by something while watching/using the animations, the question prompt list and the result page?
4. Do you have any recommendations on how to improve the animations, the question prompt list and the result page?
5. Would you like to see the animations, the question prompt list and the result page in practice?

Textbox 4. Script semi-structured interview with professionals participating in the feasibility study.

Introduction
Thank you for participating in this interview as part of the ADappt feasibility study. This interview will be recorded and transcribed. Your name will not be mentioned. After completing the analyses, the recording will be deleted. The interview will take approximately 20 minutes. You may stop the interview at any time if you wish.

You have participated in the ADappt feasibility study. The aim of this study is to assess whether ADappt can be used in the memory clinic and to evaluate its use.

1. What do you remember about the study? What comes to mind?
2. What were your experiences during the study?
3. How did you experience using ADappt in daily practice? Were you able to use ADappt?
4. How did you use ADappt?
5. ADappt consists of different modules (Shared Decision-Making, Diagnostic Tests, Prediction Tool, Topic List). Which modules did you use?
    a. Which modules did you find most useful or easy to use, and why?
    b. Which modules did you find less useful or more difficult to use, and why?
6. ADappt also includes several tools for patients. Did you have the impression that these tools were used by patients?
    a. If yes, in what way? What did you notice, and what did you think of that?
7. Who do you think benefits the most from ADappt? In your opinion, what is the added value of ADappt? In what way could its use have a positive effect?
8. What are practical barriers to using ADappt?
    a. What could help overcome these barriers?
9. Do you still use ADappt occasionally? In what way?
10. Do you have any additional experiences, tips, or comments about ADappt and its implementation in the memory clinic?

Table 1: Overview questionnaires feasibility study

**Professionals**	
Questionnaire at the beginning of the study	• Sociodemographics<br>• Professional characteristics<br>• Memory clinic characteristics
Questionnaire directly after a consultation	• Patient demographics<br>• Which diagnostic tests are/were performed<br>• Who made the decision<br>• In case of a results appointment:<br>o Syndromal + etiological diagnosis<br>o Cognitive test results<br>o What<br>• Whether ADappt was used, and if not, why<br>• If yes, which modules were used<br>• Satisfaction with the use of ADappt in that specific consultation on a single Visual Analogue Scale (VAS; 0=Not at all satisfied, 100=Very satisfied)<br>• Open-ended question for questions/remarks
Questionnaire at the end of the study	• Usability of ADappt by using the System Usability Scale [1] (SUS; 10 statements; 5-point Likert scale (1=Strongly disagree, 5=Strongly agree)<br>• Usefulness per module, e.g. "*I found the module Topic list useful*"; 5 items; 5-point Likert scale (1=Strongly disagree, 5=Strongly agree)<br>• Whether clinicians would like to use ADappt in their daily routine (1 item; 5-point Likert scale (1=Strongly disagree, 5=Strongly agree))<br>• Whether clinicians could easily integrate ADappt in their daily routine (1 item; 5-point Likert scale (1=Strongly disagree, 5=Strongly agree))<br>• Whether any issues in the use of ADappt were encountered, e.g. technical issues (4 items, with the possibility for self-thought issues; 4-point Likert scale (1=Not at all, 4=Very much)<br>• An open-ended question: *For future studies, do you think we need to make adjustments in ADappt and/or in the training?*
**Patients and care partners**	• Demographics<br>• Need for information (self-developed item – used in previous research[2]), "Some people want little information about their disease and care. Other people want a lot of information. How much information do you want about your disease and care?", 10-point scale, 0 = I want to know as little as possible, 10 = I want to know as much as possible)<br>• Preference role in decision-making<br>• Health literacy (3HL[3], 5 items, 4-point Likert scale)<br>• Appointment characteristics<br>• Satisfaction with the physician and the consultation, using the Patient Satisfaction Questionnaire[4] (PSQ – Dutch version; 5 items, 0=Not at all, 100=Very much) and a single Visual Analogue Scale (VAS; 0=Not at all satisfied, 100=Very satisfied)<br>• A selection of items of the European Organization for Research and Treatment of Cancer Quality of Life Group information questionnaire (EORTC QLQ-INFO 25), assessing patients'/care partners' perceptions and evaluation of the information they received (5-8 items; 4-point Likert scale; 1=None at all, 4=Very much), and their wishes for more/less information (2 items; no/yes, and two open-ended questions).[5]

	• Perception of the professional's SDM behavior: Nine-item Shared Decision Making Questionnaire (SDM-Q-9; six-point Likert scale ranging from 0: completely disagree to 5: completely agree)<br>• Opinions on ADappt, using study specific items:<br>o Awareness of existence of the patient tools (*yes/no*)<br>o Whether they used the tools (*yes/no*)<br>o To what extent the tools helped them to prepare for the consultation (4-point Likert scale; 1=Not at all, 2=A little, 3=Quite a bit 4=Very much)<br>o Whether they would recommend the use of the tools for other patients (*yes/no*), and why (*open-ended question*)<br>• Satisfaction with the consultation on a single Visual Analogue Scale (VAS; 0=Not at all satisfied, 100=Very satisfied)

References

1 Brooke, J. SUS : a "quick and dirty" usability scale. *Usability Evaluation in Industry*, 189-194 (1996).

2 Kunneman, M. *et al.* Patients' and caregivers' views on conversations and shared decision making in diagnostic testing for Alzheimer's disease: The ABIDE project. *Alzheimers Dement (N Y)* **3**, 314-322 (2017). https://doi.org/10.1016/j.trci.2017.04.002

3 Ishikawa, H., Takeuchi, T. & Yano, E. Measuring functional, communicative, and critical health literacy among diabetic patients. *Diabetes care* **31**, 874-879 (2008).

4 Blanchard, C. G., Ruckdeschel, J. C., Fletcher, B. A. & Blanchard, E. B. The impact of oncologists' behaviors on patient satisfaction with morning rounds. *Cancer* **58**, 387-393 (1986).

5 Arraras, J. I. *et al.* An international validation study of the EORTC QLQ-INFO25 questionnaire: An instrument to assess the information given to cancer patients. *European Journal of Cancer* **46**, 2726-2738 (2010). https://doi.org/https://doi.org/10.1016/j.ejca.2010.06.118
