# Supplementary material for: Usability and feasibility of ADappt: a digital toolkit to support communication on diagnosis and prognosis in memory clinics
Source: Alzheimers Res Ther. 2025 Oct 2;17:218. doi: 10.1186/s13195-025-01847-y (PMC12492680; doi:10.1186/s13195-025-01847-y)
Supplement: Supplementary file 2 — Supplementary Material 2 [file 13195_2025_1847_MOESM2_ESM.pdf]

Alzheimercentrum Amsterdam
Amsterdam UMC

De gesprekswijzer: Welke vragen heeft u?

U heeft een afspraak in een geheugenpolikliniek. Deze gesprekswijzer met voorbeeldvragen kunt u printen en meenemen naar uw afspraak op de poli.

Kruis hieronder de vragen aan die u wilt stellen aan de arts. Heeft u andere vragen? Onderaan de pagina kunt u deze vragen opschrijven.

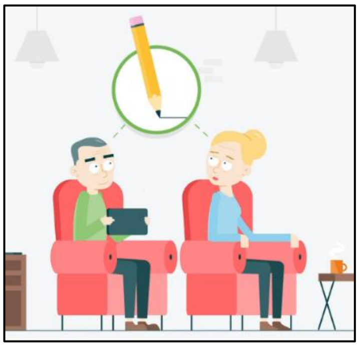

Dit wil ik vragen:

- ☐ Wat kan ik verwachten dat er in de geheugenpolikliniek gaat gebeuren?
- ☐ Welke testen of onderzoeken zijn er mogelijk?
- ☐ Wat zou er uit het onderzoek kunnen komen?
- ☐ Wat is de reden om een bepaald onderzoek wel of niet bij mij te doen?
- ☐ Wat is de uitslag van de onderzoeken die bij mij zijn gedaan? (Bijvoorbeeld een hersenscan, neuropsychologisch onderzoek, of ruggenprik)
- ☐ Wat zegt de uitslag van het onderzoek over de oorzaak van mijn klachten?
- ☐ Hoe zeker is deze uitslag?
- ☐ Wat heb ik precies en wat betekent dat?
- ☐ Heeft dit gevolgen voor mij? Bijvoorbeeld:
- ☐ Moet ik nu zorg regelen?
- ☐ Mag ik nog autorijden? Heeft dit gevolgen voor mijn rijbewijs?
- ☐ Moet ik medicijnen gaan gebruiken?
- ☐ Wat kan ik verwachten dat er gaat gebeuren in de toekomst?
- ☐ Wat kan ik zelf doen?
- ☐ Wie neemt mijn zorg na deze afspraak over?

Overige vragen:

©2020 - De gesprekswijzer is ontwikkeld door Alzheimercentrum Amsterdam
De gesprekswijzer en animaties zijn gebaseerd op de ABIDE topiclijst (te vinden op: https://www.adappt.health).
