# Supplementary material for: Usability and feasibility of ADappt: a digital toolkit to support communication on diagnosis and prognosis in memory clinics
Source: Alzheimers Res Ther. 2025 Oct 2;17:218. doi: 10.1186/s13195-025-01847-y (PMC12492680; doi:10.1186/s13195-025-01847-y)
Supplement: Supplementary file 3 — Supplementary Material 3 [file 13195_2025_1847_MOESM3_ESM.pdf]

Uitslagpagina patiënt

Uw situatie

Bij u is sprake van milde cognitieve klachten. Deze diagnose 'MCI' betekent ook dat u een verhoogde kans heeft om in de toekomst dementie te ontwikkelen.

Bij u zijn aanvullende diagnostische tests uitgevoerd. Deze tests tonen aan of er in uw hersenen al sprake is van Alzheimer-schade. Het is mogelijk om Alzheimer-schade te hebben, zonder dat er sprake is van dementie. Maar deze schade geeft wel een verhoogde kans op dementie in de komende jaren.

MRI

Een MRI is een foto van uw hersenen. Hier ziet u een voorbeeld van een MRI van gezonde hersenen. Op een MRI kunnen we zien of er sprake is van hersenkrimp (atrofie).

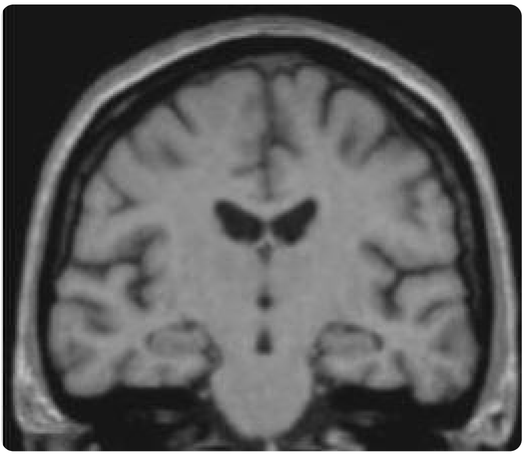

In uw geval is er mogelijk sprake van hersenkrimp. De kans om de komende jaren dementie te ontwikkelen verandert door deze informatie nauwelijks.

Duidelijk geen krimp	**Niet duidelijk of er krimp is**	Duidelijk wel krimp (atrofie)

Ruggenprik

[illegible]

Met de ruggenprik (lumbaalpunctie) tonen we aan of er aanwijzingen zijn voor de aanwezigheid van de Alzheimer-eiwitten in de hersenen.

Er zijn 2 soorten eiwitten: amyloid en p-tau.

700	24
**Amyloid**	**Tau**

Er is geen overtuigende aanwezigheid van Alzheimer-eiwitten. De kans om de komende jaren dementie te ontwikkelen verandert door deze informatie nauwelijks.

Duidelijk geen Alzheimer-eiwitten aanwezig	**Niet duidelijk of er Alzheimer-eiwitten aanwezig zijn**	Duidelijk wel Alzheimer-eiwitten aanwezig

Wat betekent dit voor u de komende tijd?

Om een uitspraak te kunnen doen over de kans dat uw klachten zich ontwikkelen tot dementie, vergelijken we uw situatie met andere vrouwelijke patiënten in onze kliniek, met dezelfde leeftijd en resultaten op diagnostische tests als u.

Van die vrouwen in dezelfde situatie als u, ontwikkelen er 3 van de 100 binnen nu en 1 jaar dementie.

Als we een langere periode bekijken, dan ontwikkelen 38 van de 100 vrouwen in dezelfde situatie als u binnen nu en 3 jaar dementie.

Op nog langere termijn, dan ontwikkelen 68 van de 100 vrouwen in dezelfde situatie als u binnen nu en 5 jaar dementie.

Hieronder ziet u een visuele weergave van de kans op het ontwikkelen van dementie voor 100 vrouwen van uw leeftijd, met dezelfde resultaten op de diagnostische tests, binnen nu en 1 jaar, 3 jaar en 5 jaar.

1 jaar

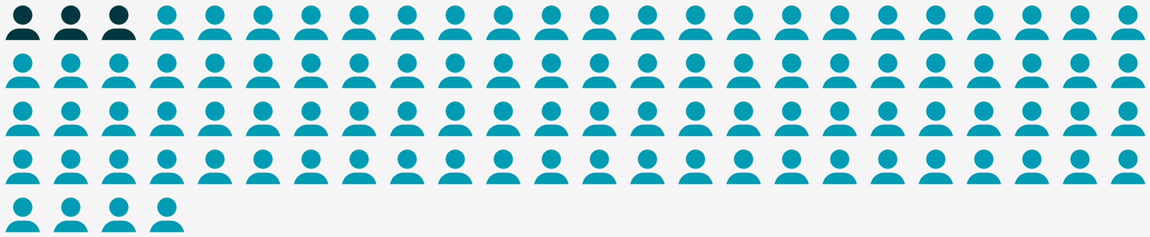

3 mensen ontwikkelen binnen nu en 1 jaar dementie.

97 mensen ontwikkelen binnen nu en 1 jaar geen dementie.

3 jaar

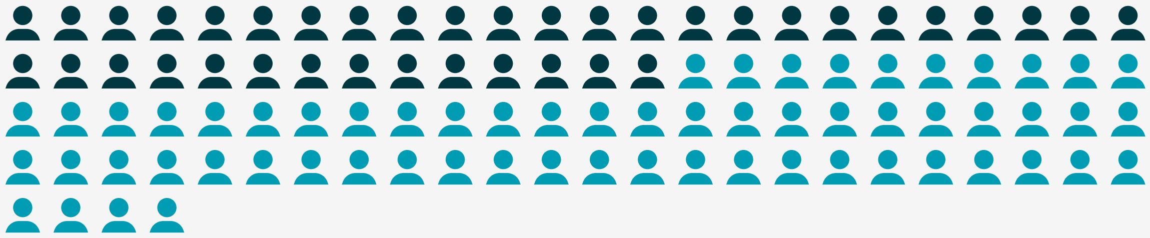

38 mensen ontwikkelen binnen nu en 3 jaar dementie.

62 mensen ontwikkelen binnen nu en 3 jaar geen dementie.

5 jaar

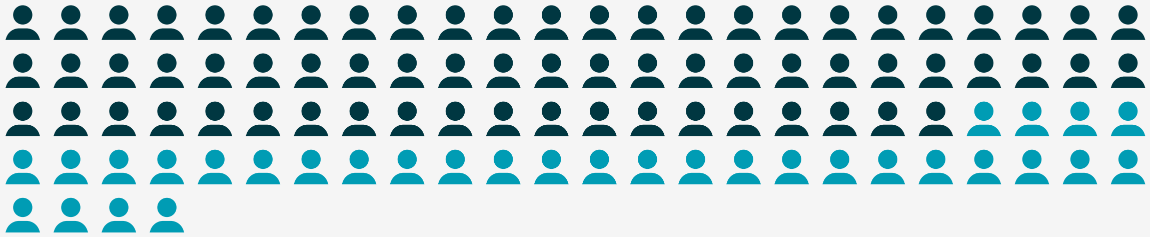

68 mensen ontwikkelen binnen nu en 5 jaar dementie.

32 mensen ontwikkelen binnen nu en 5 jaar geen dementie.
