# Supplementary material for: Usability and feasibility of ADappt: a digital toolkit to support communication on diagnosis and prognosis in memory clinics
Source: Alzheimers Res Ther. 2025 Oct 2;17:218. doi: 10.1186/s13195-025-01847-y (PMC12492680; doi:10.1186/s13195-025-01847-y)
Supplement: Supplementary file 4 — Supplementary Material 4 [file 13195_2025_1847_MOESM4_ESM.pdf]

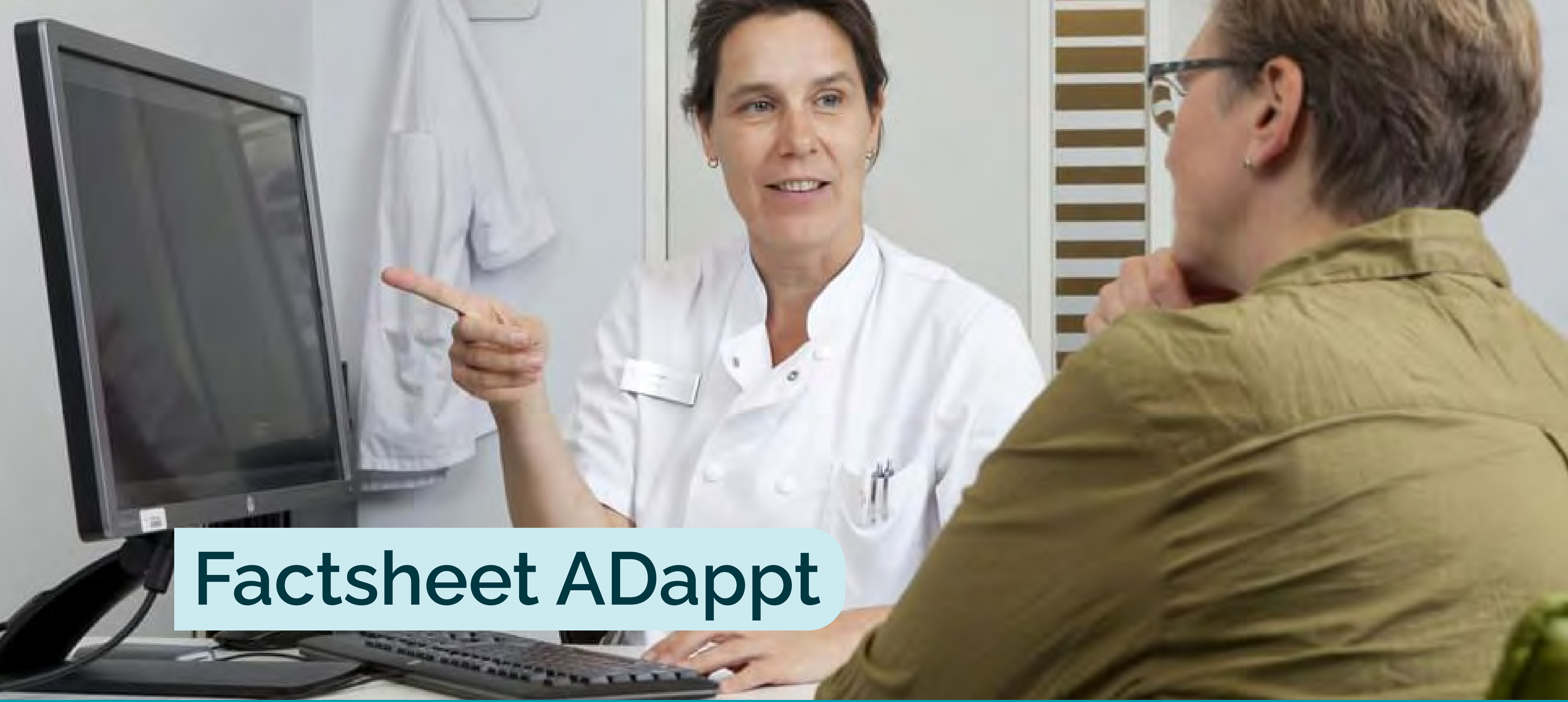

Factsheet ADappt

De online tool ADappt (www.ADappt.health) helpt professionals die werkzaam zijn op de geheugenpolikliniek om samen met patiënten en naasten te beslissen.

START

Alzheimercentrum Amsterdam
Amsterdam UMC

ABOARD
SAMEN ALZHEIMER STOPPEN VOOR HET START

Vilans

Factsheet ADappt

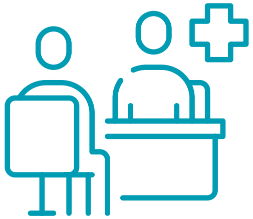

Diagnostische dilemma's in de praktijk

Dementie is een complexe aandoening, waaraan verschillende ziekten ten grondslag kunnen liggen, in de meeste gevallen de ziekte van Alzheimer. Alzheimer ontstaat geleidelijk, in de loop van vele jaren. Ontwikkeling van nieuwe diagnostische tests maakt het mogelijk om de hersenschade vast te stellen nog vóórdat er sprake is van dementie.

In de dagelijkse praktijk is het gebruik van deze diagnostische testen een uitdaging. Want wanneer gebruik je welke test? Hoe betrek je patiënten en naasten bij keuzes rondom deze diagnostiek? En hoe vertel je de uitslag aan een patiënt?

Interpretatie van de testresultaten is niet altijd eenduidig en testresultaten kunnen lastig zijn voor de patiënt en naaste om te begrijpen. Regelmatig verlaten mensen een geheugenpoli met de diagnose 'milde cognitieve stoornissen' (MCI). Een onzekere omschrijving, omdat op groepsniveau de kans op dementie dan ongeveer 50% is. Voor een betere prognose wil je zoveel mogelijk informatie halen uit de resultaten van de beschikbare diagnostische tests, zoals een MRI-scan, PET-scan of biomarkers in het hersenvocht. Je wilt niet alleen weten of er afwijkende waarden zijn, maar ook wat dit betekent voor de individuele patiënt, zodat je op individueel niveau een voorspelling kunt doen over de kans op dementie.

Het perspectief van patiënten is hierbij belangrijk. Wat verwachten zij van de diagnose? En aan welke informatie hebben zij behoefte? Het blijkt dat er vooraf weinig wordt gesproken over de verwachtingen en wensen die patiënten en hun naasten hebben over diagnostiek. Achteraf wordt er weinig gesproken over de betekenis van de uitslag en eventuele vervolgstappen. Daardoor blijven patiënten en naasten vaak met onbeantwoorde vragen achter.

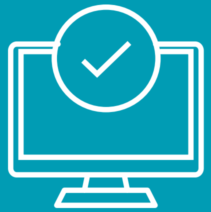

Oplossing: ADappt

Hiervoor hebben wij de online tool ADappt (www.ADappt.health) ontwikkeld. ADappt helpt professionals:

1. Om samen met patiënten en naasten te beslissen over diagnostisch testen,
2. De (biomarker) test resultaten van patiënten met de diagnose 'mild cognitive impairment' (MCI) te interpreteren,
3. Deze resultaten met patiënten en hun naasten te bespreken, en ADappt helpt patiënten en naasten
4. Om zich voor te bereiden op hun afspraken in de geheugenpolikliniek.

De modules van ADappt

1. Samen beslissen over diagnostische testen

In de **module Gespreksvijzer Samen beslissen** worden handvatten geboden voor het gesprek met de patiënt (en naaste) om samen te beslissen over het wel of niet inzetten van diagnostische testen op de geheugenpoli.
Aanvullend hierop biedt de **module Diagnostische testen** een overzicht van veelvoorkomende testen op de geheugenpoli met mogelijke voor- en nadelen van elke test. We hebben gekozen voor formuleringen die voor patiënten en naasten begrijpelijk zijn. Ook is er een animatievideo beschikbaar over de ruggenprik.

2. Hulp voor de arts bij het interpreteren van de (biomarker) test uitslagen van patiënten met de diagnose ‘mild cognitive impairment’ (MCI)

Wij hebben rekenmodellen ontwikkeld die nauwkeuriger voorspellen wat de kans is dat een individuele patiënt met MCI dementie ontwikkelt binnen één, drie of vijf jaar. Deze zijn te vinden in de **module Predictietool**. Het rekenmodel interpreteert de beschikbare uitslagen van MRI-scan, lumbaalpunctie of amyloid-PET in de context van geslacht, leeftijd en cognitieve prestaties van de patiënt.

3. Meer zekerheid over de uitslag en een uitslagpagina voor de patiënt

In de **module Uitslagpagina** kunnen deze resultaten aan de patiënt worden meegegeven. De patiënt met MCI loopt dus niet meer de geheugenpoli uit met een 50% kans op dementie, maar krijgt een uitslagpagina mee met een gepersonaliseerde prognose.

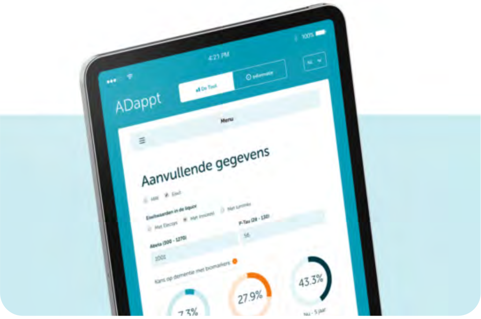

Ga naar ADappt >

4. Beter voorbereide patiënten en naasten

De **module Topic lijst** bestaat uit onderwerpen rondom diagnostiek die professionals, patiënten en hun naasten belangrijk vinden om te bespreken. Patiënten en hun naasten kunnen de lijst gebruiken ter voorbereiding op het bezoek aan de geheugenpoli, voor professionals dient de lijst als geheugensteun. Om patiënten en naasten te ondersteunen bij het voorbereiden van de consulten met hun zorgverlener in de geheugenpolikliniek vertaalden we de topic lijst voor hen in animatievideo’s over de eerste afspraak en het uitslaggesprek op de geheugenpoli. Ook maakten we een lijst met voorbeeldvragen die kan worden gedownload en uitgeprint om mee te nemen naar het gesprek. Zo worden alle belangrijke onderwerpen besproken.
