# Supplementary material for: Usability and feasibility of ADappt: a digital toolkit to support communication on diagnosis and prognosis in memory clinics
Source: Alzheimers Res Ther. 2025 Oct 2;17:218. doi: 10.1186/s13195-025-01847-y (PMC12492680; doi:10.1186/s13195-025-01847-y)
Supplement: Supplementary file 5 — Supplementary Material 5 [file 13195_2025_1847_MOESM5_ESM.pdf]

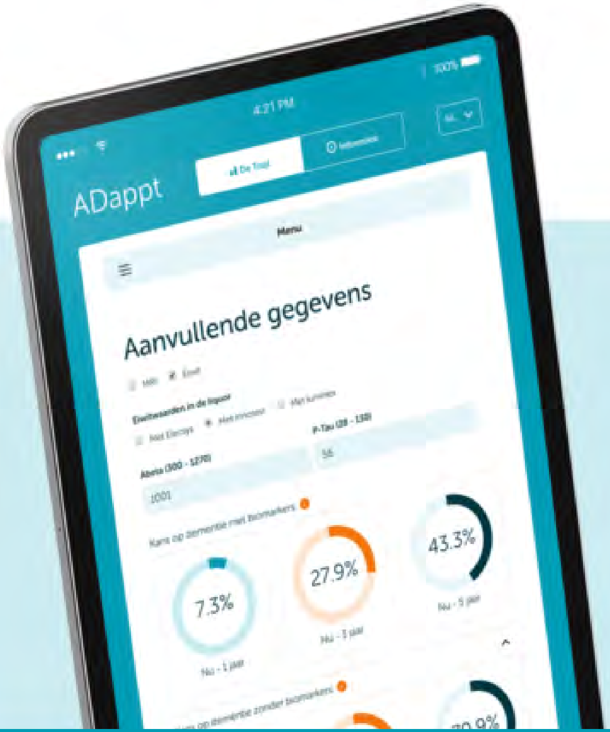

Workflow ADappt

De implementatie van ADappt vraagt aanpassingen in het dagelijkse werkproces. Hiervoor hebben we een workflow ontwikkeld. Pak deze erbij als je aan de slag gaat.

START

Alzheimercentrum Amsterdam
Amsterdam UMC

ABOARD
SAMEN ALZHEIMER STOPPEN VOOR HET START

Vilans

Workflow ADappt

De implementatie van ADappt vraagt aanpassingen in het dagelijkse werkproces. Hiervoor hebben we een workflow ontwikkeld. Pak deze erbij als je aan de slag gaat met nadenken over (het voorbereiden van) het aanpassen van je werkprocessen en het regelen van de daarbij benodigde randvoorwaarden.

De Workflow ADappt bestaat uit twee stappen:
Stap 1: Voorbereiding implementatie ADappt
Stap 2: Werkproces ADappt inrichten op de geheugenpoli

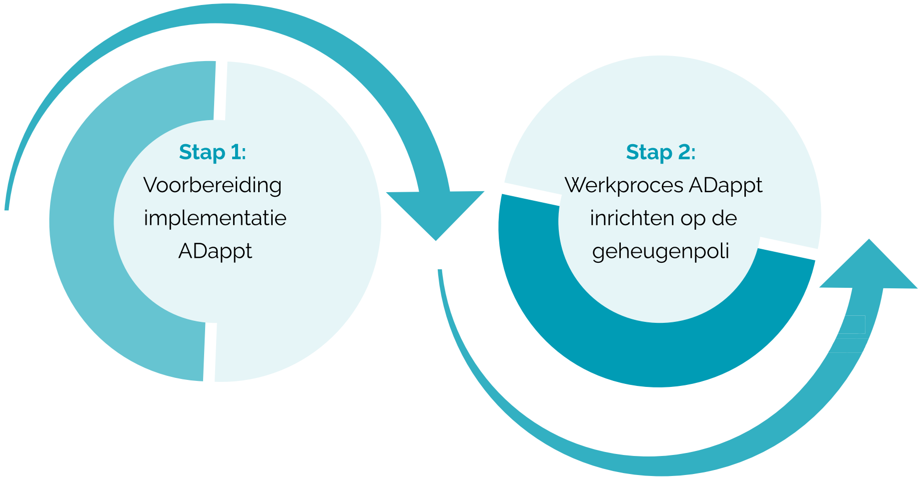

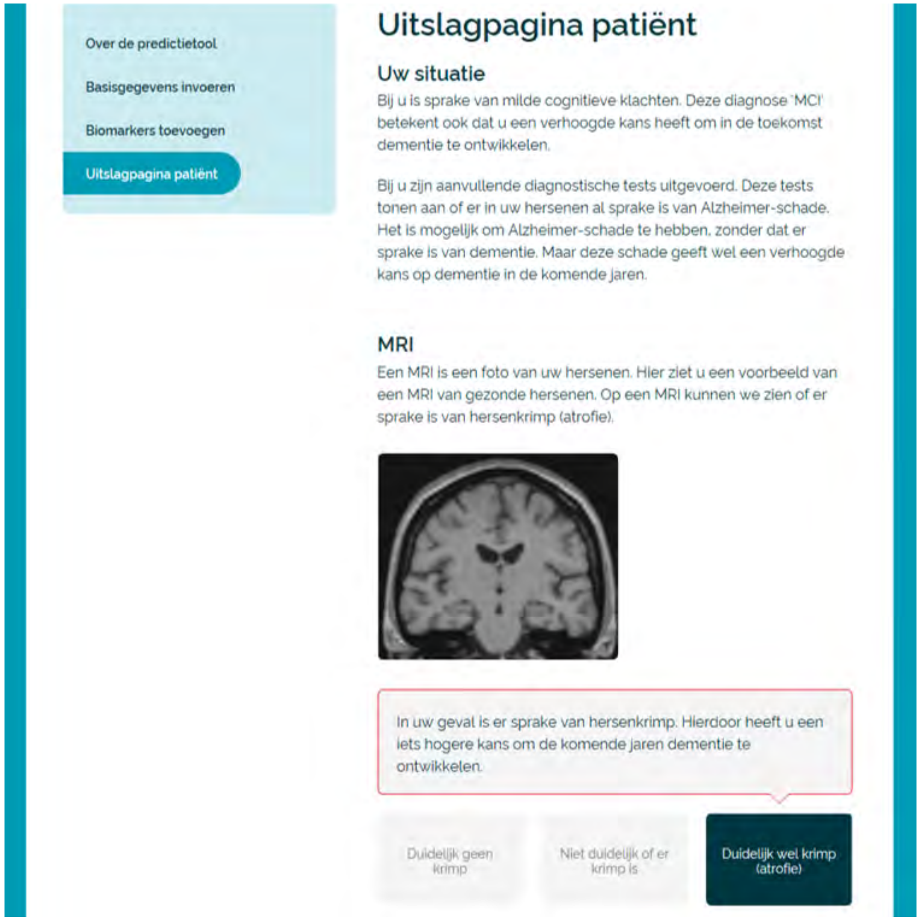

Afbeelding 1: de uitslagpagina patiënt

Stap 1:

Voorbereiding implementatie ADappt

ADappt.health is een externe website ontwikkeld voor zorgverleners, die zowel voor zorgverleners als patiënten en naasten toegankelijk is.

Module	Bedoeld voor zorgverleners	Bedoeld voor patiënten en naasten
1. Gesprekswijzer Samen beslissen	✓	
2. Diagnostische testen	✓	✓
• Video ruggenprik		✓
3. Predictietool	✓ (alleen met inlog)	
4. Patiëntvriendelijke uitslagpagina	✓	✓ (alleen via zorgprofessional; achter inlog)
5. Topic lijst	✓	✓
• Video de eerste afspraak		✓
• Video het uitslaggesprek		✓
• Lijst met voorbeeldvragen		✓

Technische aspecten:

- ADappt.health kan gebruikt worden op een pc, tablet of smartphone.
- In sommige ziekenhuizen wordt de toegang tot ADappt.health geblokkeerd door een firewall. Vraag na bij de lokale ICT afdeling of zij dit kunnen opheffen voor deze website. Als alternatief kun je ADappt.health gebruiken op je smartphone of tablet.
- ADappt.health is niet gekoppeld aan het ziekenhuisinformatiesysteem of EPD.
- ADappt.health slaat géén gegevens op. Er zijn dus geen gevoeligheden op gebied van privacy.
- De predictietool is alleen bedoeld voor zorgverleners, daarom zit deze achter een inlog. Vraag een inlog aan via https://adappt.health/nl/tool. Je ontvangt een link per mail waarmee je de predictietool opent. Voor het gemak kun je de email met inlog opslaan en de link als snelkoppeling toevoegen aan je bureaublad.
- De predictietool wordt standaard ingevuld met geslacht, leeftijd en MMSE score. Deze gegevens kunnen worden aangevuld met uitslagen van diagnostische tests (MRI, lumbaalpunctie, amyloïd-PET scan).
- De predictietool is ontwikkeld voor mensen met MCI. De rekenmodellen geven géén valide uitslagen voor mensen met subjective cognitive decline. Ook werken zij niet voor mensen met dementie.
- De patiëntvriendelijke uitslagpagina kan uitgeprint worden en meegegeven aan de patiënt of opgeslagen als PDF en gemaild naar de patiënt.
- Deze voorbeeldtekst kan worden toegevoegd aan je notitie in het EPD wanneer je de predictietool gebruikt hebt:
  - "Uitleg gegeven over prognose bij MCI m.b.v. ADappt. Uitslagpagina meegegeven"

Stap 2:

Werkproces ADappt inrichten op de geheugenpoli

Voorafgaand aan het gebruik van ADappt op de geheugenpoli:
Een lid van het ADappt team geeft training aan clinici die ADappt gaan gebruiken op hun geheugenpolikliniek.

Voorafgaand aan het 1e consult:
De secretaresse brengt patiënten op de hoogte van de hulpmiddelen die beschikbaar zijn op ADappt door middel van een informatiebrief. Dit kan per post of per mail, dat verschilt per ziekenhuis.

Informatiebrief per post:
In de informatiebrief staat uitgelegd hoe de patiënt de video “de eerste afspraak” kan bekijken en wordt de patiënt aangemoedigd om de lijst met voorbeeldvragen in te vullen en mee te nemen (wordt geprint meegestuurd).

Informatiebrief per mail:
In de informatiebrief staat een link naar de video “de eerste afspraak” en wordt de patiënt aangemoedigd om de lijst met voorbeeldvragen uit te printen, in te vullen en mee te nemen naar de afspraak.

Voorbeeld patienteninformatiebrief (aan te passen naar lay out eigen ziekenhuis)

Tijdens het 1e consult:

- Vraag de patiënt naar zijn of haar hulpvraag, eventueel aan de hand van de ingevulde lijst met voorbeeldvragen.
- Betrek de patiënt en naaste bij de besluitvorming rondom diagnostische testen met behulp van ADappt-samen beslissen en beslis samen over het inzetten van diagnostische testen.
- Geef uitleg over diagnostische tests en wat verwacht kan worden van de resultaten met behulp van ADappt-diagnostische testen.
- Check of er nog onderwerpen zijn die belangrijk zijn om te bespreken met behulp van de ADappt-Topic lijst en vraag de patiënt naar de ingevulde lijst met voorbeeldvragen. Bespreek welke vragen mogelijk nog niet zijn beantwoord.

Tijdens het MDO:

In het geval van een syndroomdiagnose MCI:

- Je hebt diagnostische testen toegepast. Gebruik de predictietool om de testresultaten te interpreteren en inzicht te krijgen in de kans dat MCI zich ontwikkelt tot dementie.

Of:

- Je overweegt aanvullende testen. Gebruik de predictietool om in te schatten hoeveel meer zekerheid een aanvullende uitslag zal geven in het geval van deze patiënt.

Voorafgaand aan het uitslaggesprek:

De secretaresse brengt patiënten op de hoogte van de hulpmiddelen die beschikbaar zijn op ADappt met een informatiebrief. Dit kan per post of per mail, dat verschilt per ziekenhuis.

Informatiebrief per post:
In de informatiebrief staat uitgelegd hoe de patiënt de video "het uitslaggesprek" kan bekijken en wordt de patiënt aangemoedigd om de lijst met voorbeeldvragen in te vullen en mee te nemen (geprint meegestuurd).

Informatiebrief per e-mail:
In de informatiebrief staat een link naar de video "het uitslaggesprek" en wordt de patiënt aangemoedigd om de lijst met voorbeeldvragen uit te printen, in te vullen en mee te nemen naar de afspraak.

Voorbeeld patienteninformatiebrief (aan te passen naar lay out eigen ziekenhuis)

In het geval van een syndroomdiagnose MCI, vult de arts de uitslagen in de predictietool in ter voorbereiding op het consult (let wel; tool slaat de gegevens niet op – eventueel tijdens consult opnieuw invullen). Eventueel kan de uitslagpagina al worden uitgeprint.

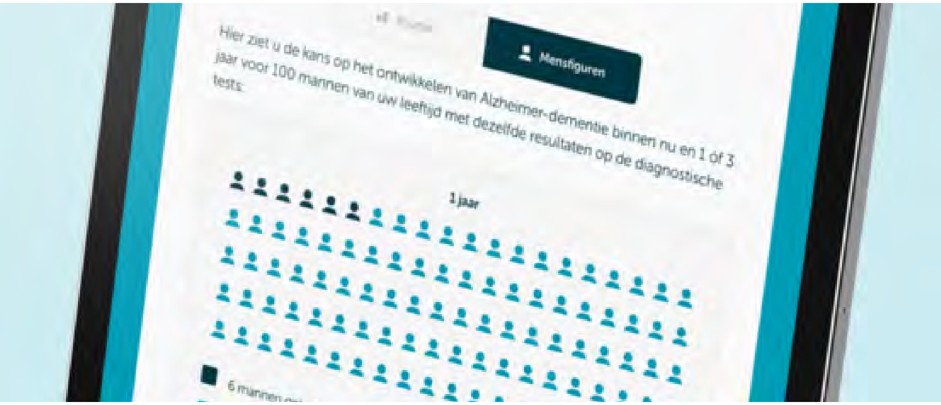

Afbeelding 2: de predictietool en de patiëntvriendelijke uitslagpagina

Tijdens het uitslaggesprek:

In het geval van een syndroomdiagnose MCI:

1. Bespreek de uitslagen aan de hand van de predictietool en de patiëntvriendelijke uitslagpagina.
2. Geef een print van de uitslagpagina mee of sla op als PDF en mail naar de patiënt.
3. Indien je aanvullende testen overweegt, kan de predictietool worden gebruikt om in te schatten hoeveel meer zekerheid een aanvullende uitslag zal geven in het geval van deze patiënt.
4. Nota bene: de predictietool geeft alleen valide resultaten voor patiënten met MCI, dus niet voor patiënten met subjectieve cognitieve achteruitgang.

Onafhankelijk van diagnose:

1. Bespreek de diagnose en vervolgstappen aan de hand van de ADappt-Topic lijst.
2. Geef uitleg over de uitslag van diagnostische testen met behulp van ADappt-diagnostische testen.
3. Pas, indien van toepassing, samen beslissen toe met behulp van ADappt-samen beslissen over het inzetten van (aanvullende) diagnostische testen.
4. Vraag de patiënt naar de ingevulde lijst met voorbeeldvragen. Bespreek welke vragen mogelijk nog niet zijn beantwoord aan de hand van de ingevulde lijst of aan de hand van de topic lijst.

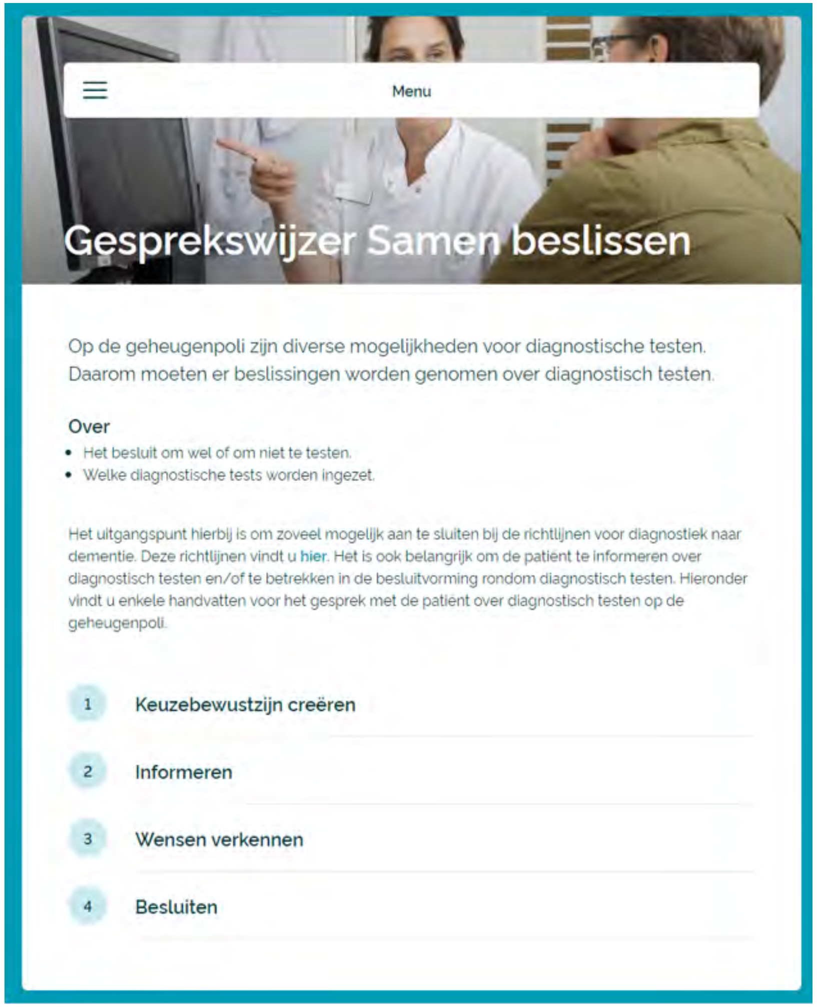

Afbeelding 3: Gesprekswijzer Samen beslissen
