# Supplementary material for: Usability and feasibility of ADappt: a digital toolkit to support communication on diagnosis and prognosis in memory clinics
Source: Alzheimers Res Ther. 2025 Oct 2;17:218. doi: 10.1186/s13195-025-01847-y (PMC12492680; doi:10.1186/s13195-025-01847-y)
Supplement: Supplementary file 6 — Supplementary Material 6 [file 13195_2025_1847_MOESM6_ESM.pdf]

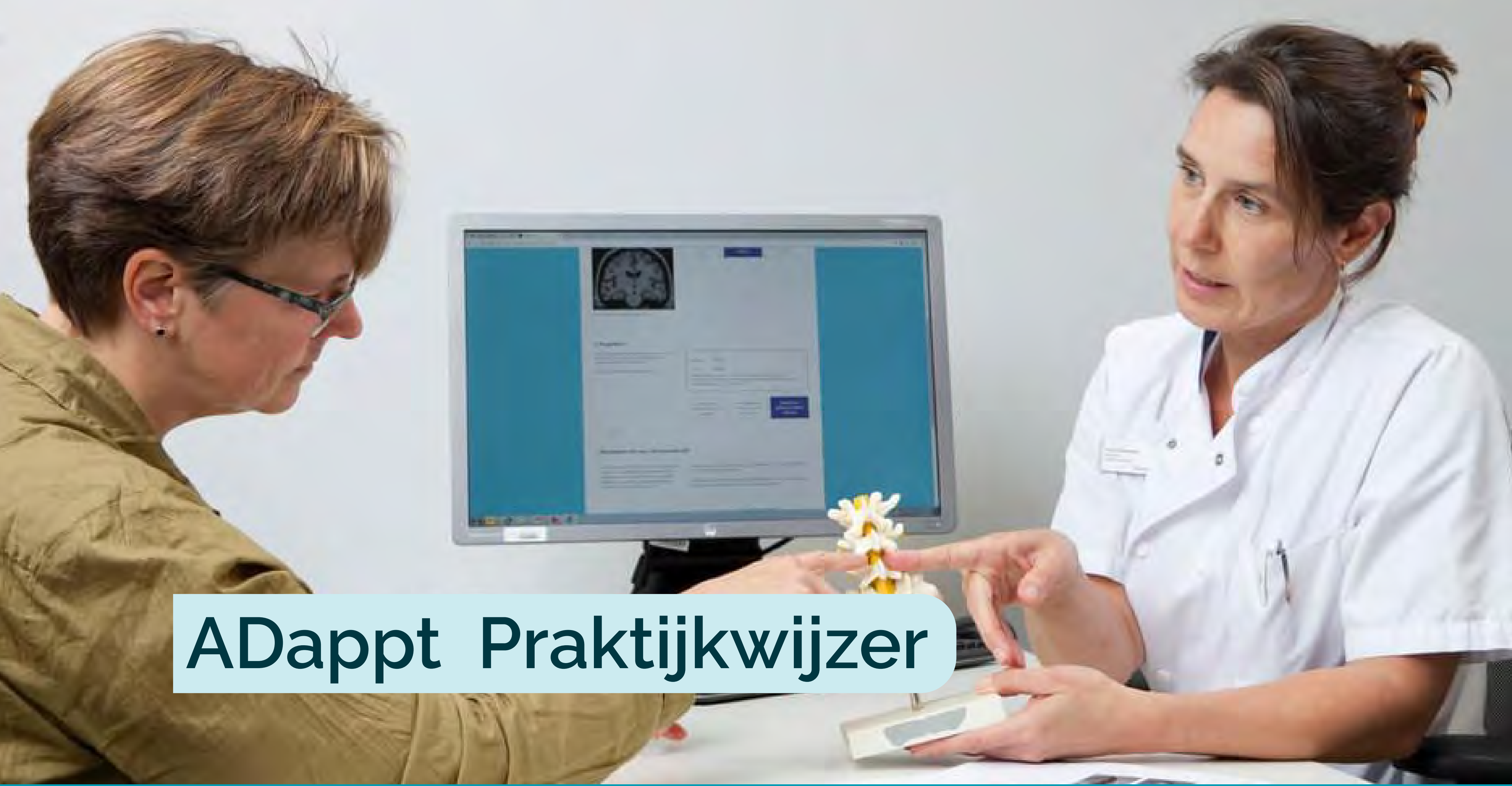

ADappt Praktijkwijzer

ADappt is een online tool voor zorgprofessionals werkzaam op de geheugenpoli. ADappt helpt professionals om samen met patiënten en naasten te beslissen.

Alzheimercentrum Amsterdam
Amsterdam UMC

ABOARD
SAMEN ALZHEIMER STOPPEN VOOR HET START

Vilans

START

ADappt Praktijkwijzer

Het aantal diagnostische mogelijkheden bij geheugenklachten is toegenomen, waardoor patienten beter geïnformeerd kunnen worden over de oorzaak en het verloop van hun klachten. Dit levert in de praktijk echter nieuwe dilemma's op: hoe speel je goed in op de individuele informatiebehoeften en verwachtingen van patiënten en naasten? Wanneer gebruik je welke test? Hoe betrek je patiënten en naasten bij keuzes rondom deze diagnostiek? Hoe interpreteer je de resultaten? En hoe vertel je de uitslag aan een patiënt? Daarom willen we op de geheugenpolikliniek aan de slag met de implementatie van ADappt!

ADappt is een online tool voor zorgprofessionals werkzaam op de geheugenpoli (www.ADappt.health). ADappt helpt professionals om 1) samen met patiënten en naasten te beslissen over diagnostische testen (module Samen beslissen), 2) belangrijke onderwerpen aan bod te laten komen tijdens het consult (module Topic lijst), 3) de (biomarker) test resultaten van patiënten met de diagnose 'mild cognitive impairment' (MCI) te interpreteren (module Predictietool), en 4) deze resultaten met patiënten en hun naasten te bespreken (module Uitslagpagina). Daarnaast zijn er aan ADappt hulpmiddelen toegevoegd die patiënten en naasten kunnen gebruiken om hun bezoeken aan de geheugenpoli voor te bereiden: i) animatievideo's over het eerste gesprek, de ruggenprik en het uitslaggesprek op de geheugenpoli, ii) een gespreksonderwerpenlijst: een lijst met voorbeeldvragen die kan worden gedownload en uitgeprint om mee te nemen naar het gesprek.

De implementatie van ADappt is een nieuwe werkwijze en heeft invloed op het dagelijks werk. Het vraagt een verandering en verankering van de werkprocessen en het gedrag van betrokkenen, zowel van zorgprofessionals als van patiënten en hun naasten. De implementatie is daarom geen koud kunstje, maar vraagt moeite en inspanning van alle betrokkenen en daarmee een goede voorbereiding en een plan van aanpak.

Je hoeft het wiel gelukkig niet helemaal zelf uit te vinden! Op basis van eerste ervaringen van zorgprofessionals, patiënten en naasten bij het gebruik van ADappt in de klinische praktijk heeft het Alzheimercentrum Amsterdam met kenniscentrum Vilans een praktijkwijzer gemaakt. Deze ADappt Praktijkwijzer bevat een opzet voor de aanpak van de implementatie, allerlei praktische hulpmiddelen, tips en voorbeelden uit de praktijk.

Ga ook aan de slag met het implementeren van ADappt en doorloop de volgende 8 implementatiefasen van Kotter

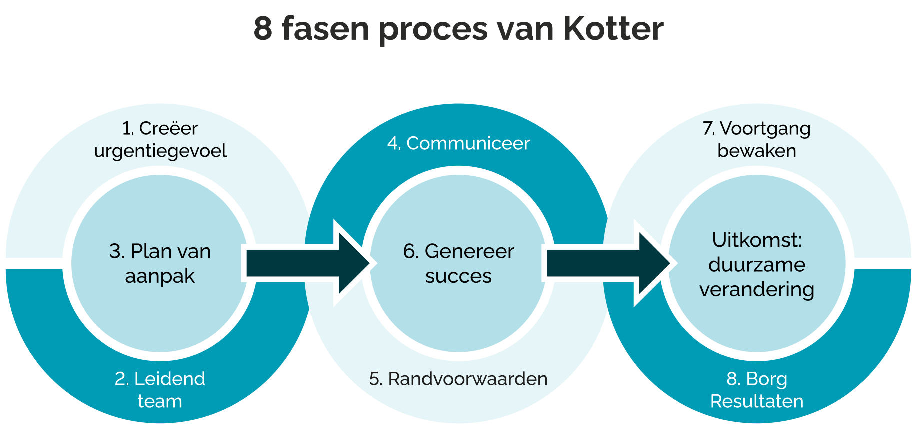

Figuur 1: Kotter, J. P. Leading Change. Boston: Harvard Business School Press, 1996.) https://www.kotterinc.com/8-steps-process-for-leading-change/

8 fasen proces van Kotter

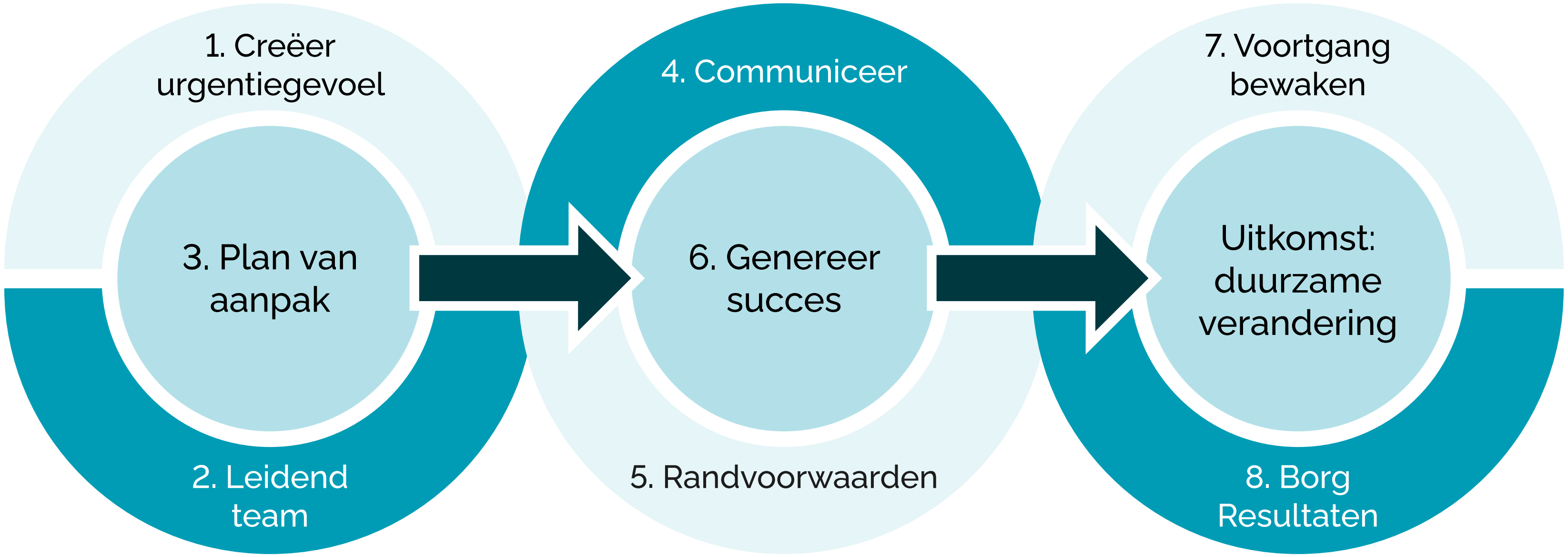

Deze **ADappt Praktijkwijzer** bevat een opzet voor de aanpak van de implementatie, allerlei praktische hulpmiddelen, tips en voorbeelden uit de praktijk.

FASE 1.
Creëer urgentiebesef

FASE 2.
Vorm een leidend team

FASE 3.
Maak een plan van aanpak

FASE 4.
Communiceer

FASE 5.
Creëer de juiste randvoorwaarden

FASE 6.
Genereer succes

FASE 7.
Bewaak de voortgang

FASE 8.
Borg de resultaten

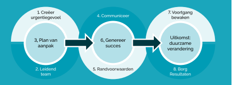

Creëer een gevoel van urgentie

Voordat ADappt geïmplementeerd kan worden en iedereen er mee aan de slag kan, is het belangrijk dat alle betrokkenen doordrongen zijn van het belang van ADappt. Bekijk daarom van tevoren welke partijen van dit belang overtuigd moeten zijn. Waarom zouden zij, juist nu, gebruik moeten gaan maken van ADappt: wat is de meerwaarde? En wat zijn oorzaken van desinteresse of weerstand? Verduidelijk hierbij ook de rol en benodigde steun van het management bij het veranderingsproces, bijvoorbeeld in het faciliteren van randvoorwaarden zoals tijd en geld. Omdat emoties de motor zijn voor verandering, helpt het om bij het overtuigen de nadruk te leggen op emoties als hoop, vertrouwen, optimisme en enthousiasme ('see-feel-change'), en niet alleen op rationele zaken als feiten en figuren ('analyse-think-change'). Een gevoel van urgentie creëer je namelijk eerder door te visualiseren, door de bril van de patiënt naar de geheugenpoli te kijken en een krachtig toekomstbeeld te schetsen, dan met een dik rapport over ontevreden patienten aan te komen.

Hulpmiddelen en tips

1. Maak gebruik van deze presentatie voor de medische staf waarmee je (het belang van het) implementeren van ADappt met hen kan bespreken. Om de vakgroep actief te betrekken zijn er diverse vragen opgenomen om samen over na te denken en over uit te wisselen. Zo kunnen zowel de kansen/voordelen als de zorgen/belemmeringen op tafel komen en worden verdiept, afgewogen en/of worden weggenomen. De presentatie kan de vakgroep zo helpen om een gedragen besluit te nemen om wel of niet aan de slag te gaan met de implementatie van ADappt op de geheugenpoli in jouw ziekenhuis.

2. Naast de vakgroep zijn belangrijke stakeholders in dit beginstadium het management (afdelingsmanagement en de Raad van Bestuur) en mogelijk al specifieke stafafdelingen (zoals bijvoorbeeld het kwaliteitsbureau, de ICT afdeling of juridische zaken). Kijk op maat in jouw ziekenhuis bij wie het relevant is om het urgentiebesef te verhogen om een go/no go te krijgen. Met deze factsheet voor het management kun je de stakeholders in het ziekenhuis informeren over, en motiveren voor, de implementatie van ADappt.

FASE 2

Vorm een leidend team

Als er een 'go' vanuit de medische staf en het management is voor de implementatie van ADappt is het belangrijk om vervolgens te bekijken wie bepalende spelers in het veranderingsproces worden. Zo is het van belang een actieve en betrokken projectleider aan te stellen die de implementatie van ADappt coördineert, in opdracht van het management. Deze projectleider moet ondersteund worden door een enthousiast projectteam. De personen in dit team hebben formele en informele invloed, beschikken over voldoende tijd en middelen, en zijn goed geworteld in de organisatie van de geheugenpoli. Er moet een duidelijke taakverdeling en werkwijze worden afgesproken met dit projectteam, en waar nodig moet ondersteuning worden geboden. De projectleider en het projectteam betrekken vervolgens ook andere relevante personen/partijen binnen het ziekenhuis en de rest van de afdeling bij de implementatie.

Hulpmiddelen en tips

1 Stel een coördinator van het projectteam aan, de projectleider:

- Het projectteam wordt aangestuurd door een projectleider. Wie dit het beste kan zijn, is afhankelijk van de lokale situatie. Dit kan een arts zijn, maar dit hoeft niet.
- Zorg ervoor dat de projectleider voldoende tijd krijgt voor zijn/haar rol. Gemiddeld moet de projectleider 2 uur in de week gedurende 3 maanden beschikbaar zijn om de implementatie te kunnen coördineren.
- Zie verder ook de profielkenmerken in deze voorbeeld vacaturetekst projectleider

2 De projectleider stelt een, bij voorkeur klein en multidisciplinair, projectteam samen:

- Het projectteam is trekker van het implementatieprogramma binnen het ziekenhuis. Dit team bestaat uit ongeveer vier personen. Naast de projectleider gaat het vaak om een arts, een (onderzoeks)verpleegkundige en een afdelingssecretaresse/baliemedewerker.
- Het projectteam heeft de functie om als ambassadeur op te treden voor ADappt en voert het implementatieprogramma uit op locatie. Denk hierbij aan het opstellen en uitvoeren van het plan van aanpak en het helpen inbedden in de dagelijkse werkprocessen. Maar denk ook aan het informeren en enthousiasmeren van collega's binnen en buiten de afdeling (ICT, communicatie, management, patiënten-adviesraad,

etc.). Het organiseren en uitvoeren van deze activiteiten kost tijd. Zorg er dan ook voor dat alle medewerkers in dit team beschikken over voldoende tijd om deze taken uit te kunnen voeren.

3 Besteed bij de start van het projectteam aandacht aan de opdracht, taakverdeling en werkwijze van het projectteam:

- Wat hebben we als het projectteam te doen?
- Hoe vaak en wanneer komen we bij elkaar?
- Wie doet wat?
- Wat zijn de verwachtingen?
- Wie zijn belangrijke betrokkenen en belanghebbenden (denk ook aan patiënten en hun naasten)? Hoe betrekken we die (bijv. middels een stuurgroep, klankbordgroep, etc.)?

Maak een plan van aanpak: ontwikkel een visie en strategie

Als het urgentiebesef aangewakkerd is en er een projectteam is samengesteld, maakt het projectteam een plan van aanpak. Het gaat bij de implementatie van ADappt niet alleen om de 'harde'/praktische kant van de verandering (wat moeten we (anders gaan) doen), maar vooral om de 'zachte'/gedragskant (waarom willen we dat doen en hoe krijgen we betrokkenen mee om het ook echt blijvend te gaan doen). Alleen als aan deze tweede invalshoek ook voldoende aandacht wordt besteed, lukt het om tot blijvende verandering te komen. Het is daarom belangrijk om in deze fase de visie op de gewenste verandering concreet, realistisch en eenvoudig te maken. Zoek naar de essentie voor jouw geheugenpoli: om welke verandering gaat het, waarom wil de buitenwereld die verandering van ons en waarom willen we het zelf?

Hulpmiddelen en tips

1 Besteed bij het maken van je plan van aanpak aandacht aan de bovengenoemde 'zachte' en 'harde' invalshoek van de gewenste verandering. Hieronder vind je een format voor een plan van aanpak waarin deze twee invalshoeken zijn opgenomen. Zo kun je met je projectteam gemakkelijk de benodigde interventies bepalen. Heb je zelf een format voor een plan van aanpak wat je prettig vindt? Gebruik het format wat wij in deze ADappt Praktijkwijzer hebben bijgesloten dan vooral om te brainstormen met je projectteam over de inhoud van het plan van aanpak en leg een en ander daarna vast in je eigen format.

2 Op basis van de implementatie-ervaringen van de pilotziekenhuizen hebben we een voorbeeld workflow ontwikkeld. Pak deze erbij als je binnen het plan van aanpak concreet aan de slag gaat met nadenken over (het voorbereiden van) het aanpassen van je werkprocessen en het regelen van de daarbij benodigde randvoorwaarden.

3 Daarnaast zijn er 2 Infomercials beschikbaar die de implementatie ondersteunen:

1. Wat is ADappt? Doelstelling, ontwikkeling, achtergrond, meerwaarde, ervaringen.
2. Hoe gebruik je ADappt op de poli? Instructies/tips voor zorgprofessionals (gebruik van ADappt voor en tijdens consulten) en ADappt hulpmiddelen voor patiënten en naasten (ter voorbereiding op polibezoek)

Communiceer en creëer een gevoel van veiligheid

Duidelijkheid omtrent aanstaande veranderingen is belangrijk. Communiceer daarom de verandervisie die je in de vorige fase hebt geformuleerd tijdig naar de medewerkers op de afdeling, zodat ze weten waar ze aan toe zijn.

Hulpmiddelen en tips

1 Het is belangrijk dat alle relevante betrokken partijen worden meegenomen in de communicatie. Maak daarom zo vroeg mogelijk een communicatieplan voor de implementatie van ADappt in je eigen ziekenhuis. Bepaal welke verschillende doelgroepen/ partijen relevant zijn voor de communicatie. En bepaal wanneer je wat communiceert (gekoppeld aan mijlpalen of belangrijke elementen), naar welke partijen (intern en extern) en met welke communicatiemiddelen en/of -activiteiten. We hebben een format voor een communicatieplan gemaakt waarin deze verschillende aspecten zijn opgenomen. Schakel eventueel een collega van de afdeling communicatie van je eigen ziekenhuis in om je hierbij te adviseren. Heb je zelf een format voor een communicatieplan wat je prettig vindt? Gebruik het format wat wij hebben bijgesloten dan vooral om te brainstormen met het projectteam over de inhoud van het communicatieplan en leg een en ander daarna vast in je eigen format.

2 Daarnaast zijn er enkele zaken waar je alert op moet zijn:

- Zorg voor de beschikbaarheid van een inhoudelijke toelichting ten aanzien van de keuzes die zijn gemaakt in de vorm en inhoud van de implementatie-aanpak. Beargumenteer hierbij ook hoe deze keuzes bijdragen aan het realiseren van de doelen uit de visie. Zo worden de betrokken partijen ook inhoudelijk meegenomen.
- Richt een goed systeem in waar mensen terecht kunnen bij vragen. Wijs bijvoorbeeld één persoon aan waar mensen

terecht kunnen bij vragen, of maak gebruik van een speciaal e-mailadres.

- Besteed aandacht aan communicatie rondom mijlpalen en opbrengsten. Leg deze vast in nieuwsberichten en andere aansprekende communicatievormen.
- Sluit bij de communicatie naar de verschillende doelgroepen aan in taal. Voor zorgprofessionals gebruik je weer andere woorden dan voor patiënten.
- Zijn er zaken waar over gecommuniceerd moet worden? Bekijk dan of bestaande overlegstructuren kunnen worden gebruikt om betrokkenen hierover te informeren.
- Maak ook gebruik van al bestaande communicatiekanalen, bijvoorbeeld een reeds bestaande nieuwsbrief van je afdeling of krant van je ziekenhuis. Dit kost minder tijd en zo bereik je gemakkelijk en snel een grote groep mensen.
- Vergeet de communicatie en toelichting naar patiënten en naasten niet! Ook zij moeten worden meegenomen in de nieuwe werkwijze. Hiervoor kun je ook de animaties voor patiënten op ADappt.health gebruiken.

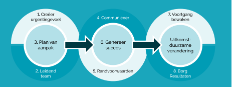

Creëer de juiste randvoorwaarden, verwijder obstakels

Om je verandering te laten slagen is onvoorwaardelijke en langdurige steun nodig. Ontwikkel daarom draagvlak voor de verandering. De belangrijkste randvoorwaarde is hierbij commitment vanuit de top: het (medisch)management van de afdeling, de Raad van Bestuur en (afhankelijk van de situatie van je ziekenhuis) stafmanagement (ICT en kwaliteit). Maar vergeet ook het draagvlak onder de verpleegkundigen, secretaresses en baliemedewerkers niet als de implementatie ook hun werkprocessen en dagelijks werkgedrag beïnvloedt. Daarnaast is het belangrijk barrières weg te nemen die het veranderingsproces bemoeilijken of in de weg staan. Denk hierbij aan belemmerende factoren voor medewerkers zoals niet communicerende ICT-systemen.

Hulpmiddelen en tips

1. Bij fase 1 heb je reeds gewerkt aan het verhogen van het urgentiebesef bij de top. Hou dat vast! Hou hen goed op de hoogte van de voortgang en neem hen op als belangrijke doelgroep in je communicatieplan in fase 4. Grijp in gesprekken en bijeenkomsten terug naar de geformuleerde visie op de verandering (zie je plan van aanpak) met de gemeenschappelijke waarden en onderliggende veronderstellingen die er aan ten grondslag liggen. En schroom ook niet om hen bewust in te schakelen en te betrekken door punten te benoemen waarvan zij persoonlijk of als groep hebben aangegeven belang aan te hechten om voortgang of doorbraken te realiseren.

2. Het verschilt per ziekenhuis in hoeverre de implementatie van ADappt andere disciplines, zoals de (onderzoeks)verpleegkundigen, baliemedewerkers of neuropsychologen, 'raakt'. Zij kunnen echter een belangrijk aandeel hebben in het implementeren van ADappt. Daarbij kan het zo zijn dat zij wel de lasten (bv. extra (registratie-) handelingen), maar niet direct de lusten (bv. dat patiënten tevredener zijn over gemaakte keuzes) ervaren. Als ze onvoldoende zijn meegenomen in de visie op de verandering, kan dat het draagvlak voor implementatie verlagen. Als de implementatie het werk van verpleegkundigen of andere professionals in jouw ziekenhuis 'raakt', is het verhogen van draagvlak bij hen dan ook cruciaal. Dat gaat dan verder dan informeren. Organiseer ook voor hen dan een aparte bijeenkomst over het project om hun urgentiebesef te verhogen en daarbij expliciet te vragen wat voor hen bij de implementatie te winnen is, danwel welke obstakels moeten worden weggenomen

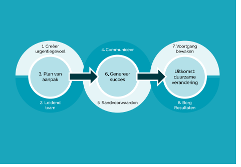

FASE 5

3 Neem belemmerende factoren die het veranderingsproces bemoeilijken of in de weg staan serieus en neem proactief activiteiten op in je plan van aanpak in fase 3 om deze te weg te nemen

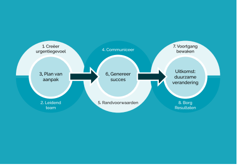

Creëer korte termijn successen

Het bepalen van mijlpaalmomenten en aandacht besteden aan korte termijn successen ('quick wins') zijn belangrijk voor de motivatie om door te gaan met de verandering. Zeker als het veranderingsproces een langere periode beslaat. Een positieve benadering van de verandering enthousiasmeert degenen die hiermee te maken hebben en creëert meer vertrouwen in de verandering. Daarnaast is het een beloning voor de medewerkers die hard werken om ADappt op de afdeling te implementeren.

Hulpmiddelen en tips

1. In deze fase ben je volop bezig met activiteiten vanuit het plan van aanpak.

2. Definieer als projectteam duidelijke mijlpaalmomenten bij verschillende stappen of onderdelen in je plan van aanpak. Bv. 'het projectteam is samengesteld en gestart', 'de kick off is geweest', 'de techniek voor het werken met ADappt staat klaar', 'de predictietool van ADappt is voor de eerste patiënt ingevuld', 'de 1e uitslagpagina is met patiënten besproken', etc.

3. Als de verschillende stappen of onderdelen veel tijd in beslag gaan nemen, is het voor de motivatie van betrokkenen belangrijk om de mijlpalen eventueel in kleinere deelstappen te definiëren.

4. Maak de mijlpaalmomenten van het project inzichtelijk voor iedereen, zodat iedereen weet waar jullie zitten in het proces en dat het project gestart is met voorbereidingen, ook als iemand van de afdeling daar in een bepaalde fase (bv. de technische voorbereiding of het ontwerp van de werkprocessen) niet zelf direct bij betrokken is. Maak het letterlijk inzichtelijk en hang de planning met de mijlpaalmomenten of de voortgang op een specifieke mijlpaal op in jullie kantoor.

5. Besteed aandacht aan de korte termijn successen in je communicatie-uitingen en -activiteiten naar de verschillende doelgroepen. Hou eventueel een 'gouden boekje' bij met positieve

ervaringen van patiënten en zorgverleners. Laat betrokkenen zelf aan het woord over hun ervaringen in de communicatie-uitingen. Dat maakt hen nog meer gezicht en eigenaar van de gewenste verandering.

6 Plan met elkaar momenten en acties om korte termijn successen letterlijk te vieren. Dat hoeft niet altijd groots, maar kan al met kleine gebaren: iets lekkers bij de koffie, een persoonlijk kaartje, etc. Deze 'vier'-momenten geven je meteen gelegenheid om mensen weer even mee te nemen in de voortgang.

7 Maak het leuk en gezellig. Gebruik humor. De positiviteit die daar uit voortkomt, maakt dat betrokkenen sowieso graag bij de ingezette beweging horen en graag mee willen doen. Denk daarom naast de inhoud veel na over de vorm waarin je zaken giet: hoe kun je het leuker of pakkender maken? Maak je een 'droge' presentatie of een met plaatjes en af en toe leuk filmpje er tussen. Laat je bij een informatiebijeenkomst de voortgang in een staatje zien of maak je een quiz over de voortgang en degene met de meeste goede antwoorden krijgt een prijs. Tip: Ontwerp eens een eenvoudige kahoot-quiz om na een wat uitgebreidere (informatie)bijeenkomst te kijken of alle medewerkers het proces in hun hoofd hebben en start klaar zijn.

FASE 6

Bewaak de voortgang, consolideer de verandering

Zodra de eerste korte termijn successen zich voordoen, is het zaak dat je afdeling niet overmoedig wordt en het gevoel krijgt dat het veranderingsproces al bijna voltooid is. Om het werkgedrag blijvend te veranderen en de nieuwe werkwijze te verankeren is vaak een langere periode en verdieping nodig. Onderzoek wat wel en niet werkt in de praktijk en verfijn de aanpak.

Je zit als medewerker vaak in een tussen- of overgangsfase: sommige werkzaamheden doe je op de oude en sommige op de nieuwe manier. Doordat het proces van implementatie vaak nog uitproberen en experimenteren is, loopt nog niet alles zoals het zou moeten. Een belangrijke valkuil is dan ook vermoeidheid of frustratie.

In deze fase ligt de nadruk op het door- en voortzetten van de verandering, afstemming en fine-tuning. Hiervoor moet het urgentiebesef op peil blijven. Het blijvend benadrukken van het belang en de meerwaarde van ADappt moet daarom hoog op de agenda staan.

Hulpmiddelen en tips

1. Evalueer regelmatig de voortgang met betrokkenen: wat gaat goed en wat kan beter? Maak eventueel gebruik van een logboekje waarin medewerkers en patiënten hun ervaringen van die dag meteen kunnen opschrijven. Daarmee kun je snel en laagdrempelig informatie verzamelen (zonder dat mensen daarvoor speciaal naar een bijeenkomst moeten komen) en eventuele aanpassingen doen.

2. Neem ervaren belemmeringen serieus en onderneem acties om deze weg te nemen.

3. Blijf benoemen waarom jullie het doen: haal regelmatig jullie aan het begin geformuleerde visie op de verandering aan.

4. Evalueer ook regelmatig als projectteam: stel eventueel je plan van aanpak bij of formuleer extra (zachte) activiteiten om de voortgang te bevorderen.

5. Meten is weten! Om de implementatie goed te kunnen monitoren over wat langere termijn, is het belangrijk om een aantal procesmaten bij te houden zoals bijvoorbeeld:
   - Aantal keren dat ADappt gebruikt is door zorgprofessionals, patiënten en/of naasten
   - Hoe vaak de ADappt uitslagpagina is besproken in het consult met patiënten en naasten
   - etc.

FASE 7

6 Daarnaast wil je vast kunnen stellen of je met de implementatie jullie gewenste doel bereikt. Bijvoorbeeld:

- het doel van de implementatie van ADappt is om onze patiënten d.m.v. ADappt zich beter te laten voorbereiden op het gesprek. Dit kun je bijvoorbeeld meten met vragen als:
  - In hoeverre hebben de videoanimaties en/of de gespreksonderwerpenlijst u geholpen om u voor te bereiden op het gesprek met de artsr?
  - In hoeverre hebben de videoanimaties en/of de gespreksonderwerpenlijst u geholpen om in het gesprek met de zorgverlener de dingen te bespreken die voor u belangrijk zijn?
  - In hoeverre hebben de videoanimaties en/of de gespreksonderwerpenlijst u geholpen om in het gesprek samen met de zorgverlener beslissingen te nemen over het diagnostisch testen of uw zorg?
- het doel van de implementatie van ADappt is om de diagnostiek en onze informatievoorziening op de geheugenpoli beter af te stemmen op de individuele patiënt, en diens voorkeuren, hulpvraag en informatiebehoefte.. Dit kun je bijvoorbeeld meten met vragen als:
  - In hoeverre is er voorzien in uw informatiebehoefte?
  - Had u meer informatie gewild? Waarover?
  - Had u minder informatie gewild? Waarover?

7 Bepaal welke zaken jullie bij de implementatie willen meten en monitoren en hoe jullie deze informatie het gemakkelijkste kunnen bijhouden, bijvoorbeeld of ADappt gebruikt is en welke informatie met patiënten en naasten is gedeeld. Leg vast wie verantwoordelijk is voor welke gegevens en hoe dit wordt bijgehouden.

8 Maak eventueel een poster waarop je deze gegevens bijhoudt zodat alle betrokkenen deze kunnen zien. Koppel er eventueel specifieke streefwaardes aan die je wil benutten als behaald succes (zie hierover ook fase 6).

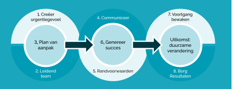

Borg de verandering, veranker de resultaten

Zorg voor bestendiging van de nieuwe situatie door veranderingen op de geheugenpolikliniek te verankeren. De verandering is geborgd als deze periodiek en systematisch wordt gemeten, geëvalueerd en bijgestuurd. Het gaat hierbij om systemen, maar vooral ook om de discipline en het doorzettingsvermogen om dingen daadwerkelijk anders te doen. In de praktijk blijft dit vaak achterwege, waardoor veranderingen verzanden. Wat niet gemeten en geëvalueerd wordt, is immers niet belangrijk genoeg.

Hulpmiddelen en tips

1 Deze laatste essentiële fase wordt vaak verwaarloosd of zelfs vergeten, waardoor de kans groot is dat de gerealiseerde verandering waar iedereen zich zo voor heeft ingespannen na verloop van tijd toch niet beklijft en betrokkenen terugvallen op hun oude werkgedrag en werkwijze.

2 Het gaat zowel om de borging van de verandering als over het nadenken over eventuele opschalingsmogelijkheden. Dit gebeurt niet vanzelf en vraagt inspanning. Zie het als projectteam dan ook echt als een extra, derde invalshoek voor je plan van aanpak die je mee programmeert en waar je acties op uitzet. In deze ADappt Praktijkwijzer vind je een format voor aanvulling van je plan van aanpak ten aanzien van de borging en opschaling. Je kunt hiermee met je projectteam de benodigde extra interventies/activiteiten bepalen voor jullie eigen ziekenhuis.

3 Ten aanzien van de borging:

- Agendeer dit onderwerp een half jaar na je start met de implementatie van ADappt. Begin op dat moment met nadenken hoe je de verandering structureel gaat inbedden.
- Denk bijvoorbeeld aan: het blijvend scholen en inwerken van nieuwe medewerkers in het gebruik van ADappt, het blijvend bijscholen van huidige medewerkers, het inbouwen in je EPD en je reguliere anamnese (voor zover je dat nog niet gedaan hebt), het inbouwen in de lokale kwaliteitscyclus, het maken van een koppeling met kwaliteit en veiligheid (bijvoorbeeld in audits), het

FASE 8

in externe visitaties laten terugkomen.

4 Ten aanzien van mogelijkheden voor opschaling:

- Agendeer dit onderwerp een jaar na je start met de implementatie van ADappt. Begin op dat moment met nadenken hoe je de verandering eventueel verder kunt opschalen:
  - Hoe kun je voor jouw doelgroep deze mooie verbetering verder brengen binnen de geheugenpolikliniek, binnen je ziekenhuis naar gelieerde specialismen dan wel in de keten? Waar liggen kansen in jouw situatie en wat is daar voor nodig? Vergeet bij het nadenken daarover niet weer opnieuw te beginnen met fase 1: het creëren van urgentiebesef.
  - Hoe lever je eventueel een bijdrage aan de landelijke opschaling op de geheugenpoliklinieken? Sluit aan bij bestaande structuren (van o.a. de NGN, NVN, NVKG) om te leren en verbeteren als geheugenpoli's in Nederland: waar kun je uitwisselen, waar deel je leerervaringen?
