# Supplementary material for: Usability and feasibility of ADappt: a digital toolkit to support communication on diagnosis and prognosis in memory clinics
Source: Alzheimers Res Ther. 2025 Oct 2;17:218. doi: 10.1186/s13195-025-01847-y (PMC12492680; doi:10.1186/s13195-025-01847-y)
Supplement: Supplementary file 7 — Supplementary Material 7 [file 13195_2025_1847_MOESM7_ESM.pdf]

ABOARD
SAMEN ALZHEIMER STOPPEN VOOR HET START

Amsterdam UMC
Universitair Medische Centra

Alzheimercentrum Amsterdam
Amsterdam UMC

Vilans

Samen beslissen rondom alzheimerdiagnostiek: ADappt.health

Handleiding

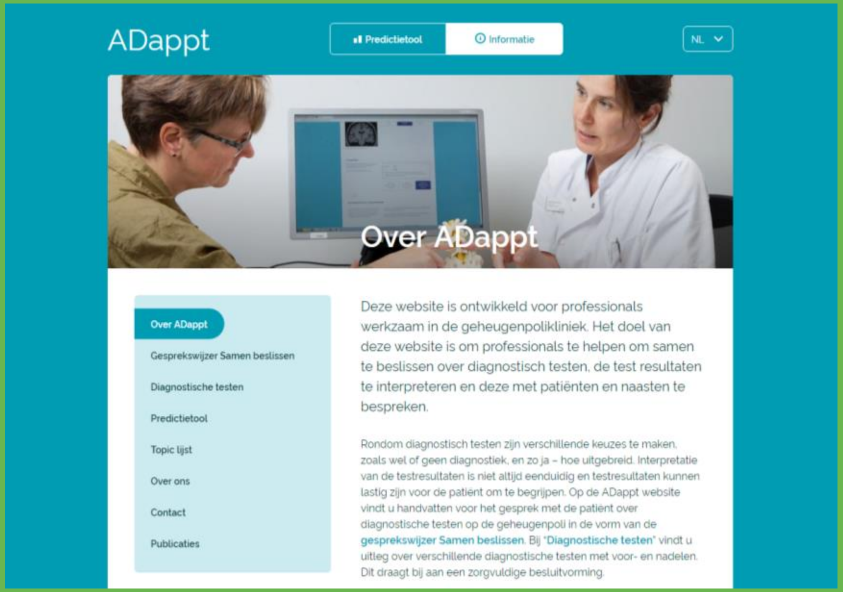

ADappt – www.ADappt.health

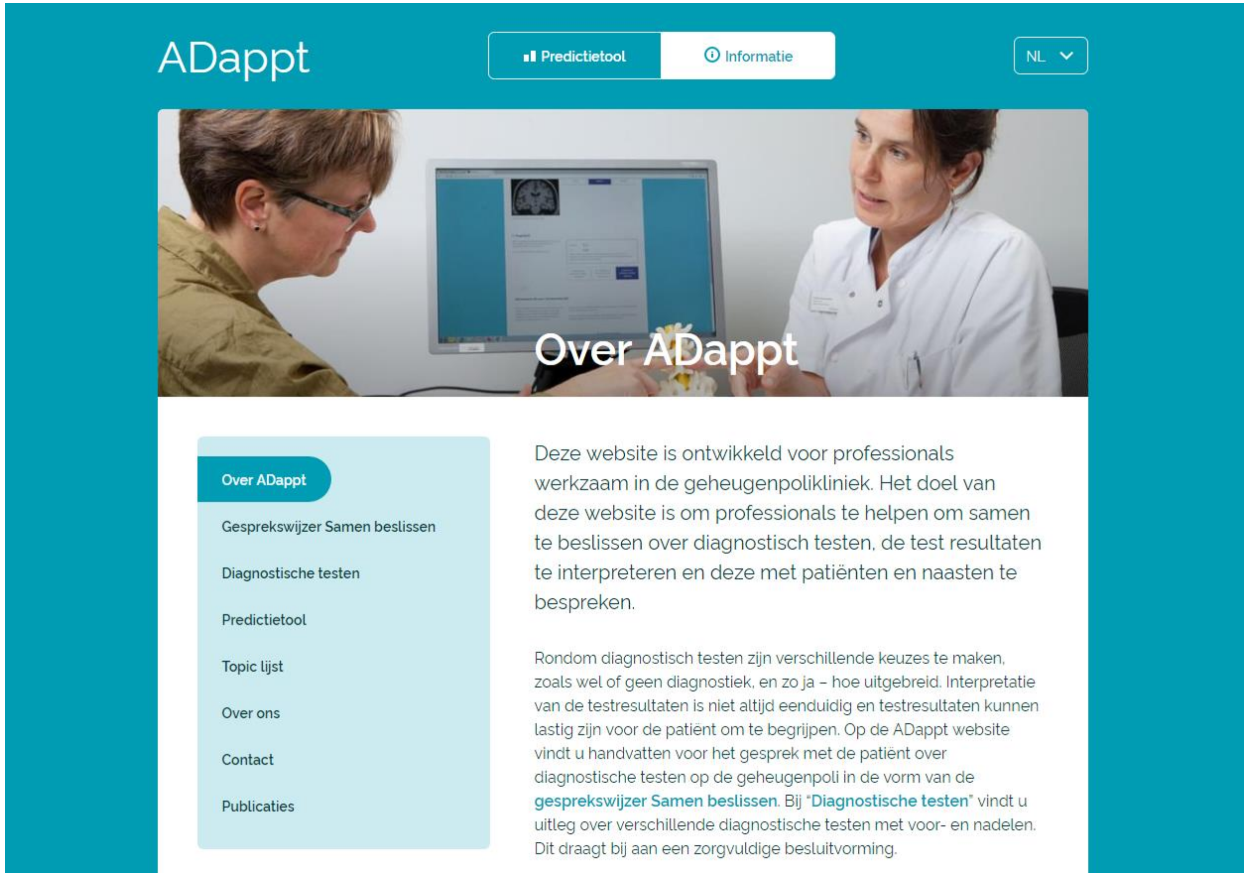

van Maurik, I. S., Visser, L. N., Pel-Littel, R. E., van Buchem, M. M., Zwan, M. D., Kunneman, M., ... & van der Flier, W. M. (2019). Development and usability of ADappt: Web-based tool to support clinicians, patients, and caregivers in the diagnosis of mild cognitive impairment and alzheimer disease. *JMIR formative research*, *3*(3), e13417.

Casus mw. Van Dijk - huisarts

- 70-jarige vrouw
- Sinds twee jaar toenemende klachten van vergeetachtigheid
- MULO, directie secretaresse
- Gaan geen grote dingen mis
- Zorgen gesterkt door moeder die op 78-jarige leeftijd

de diagnose dementie door de ziekte van Alzheimer kreeg

- MMSE 26/30

Huidige patiëntroute

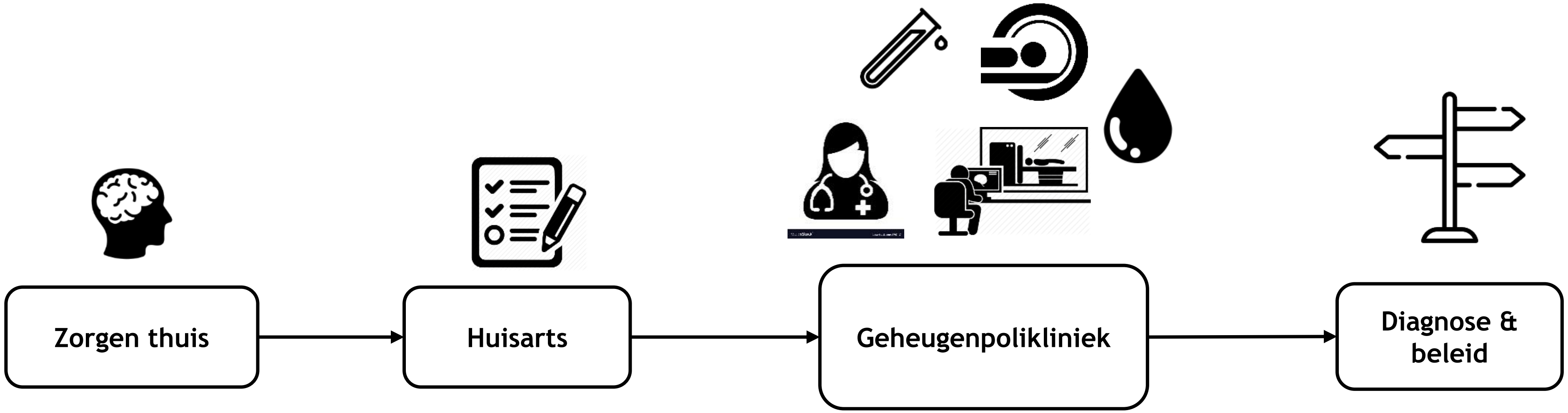

Patiëntroute met ADappt

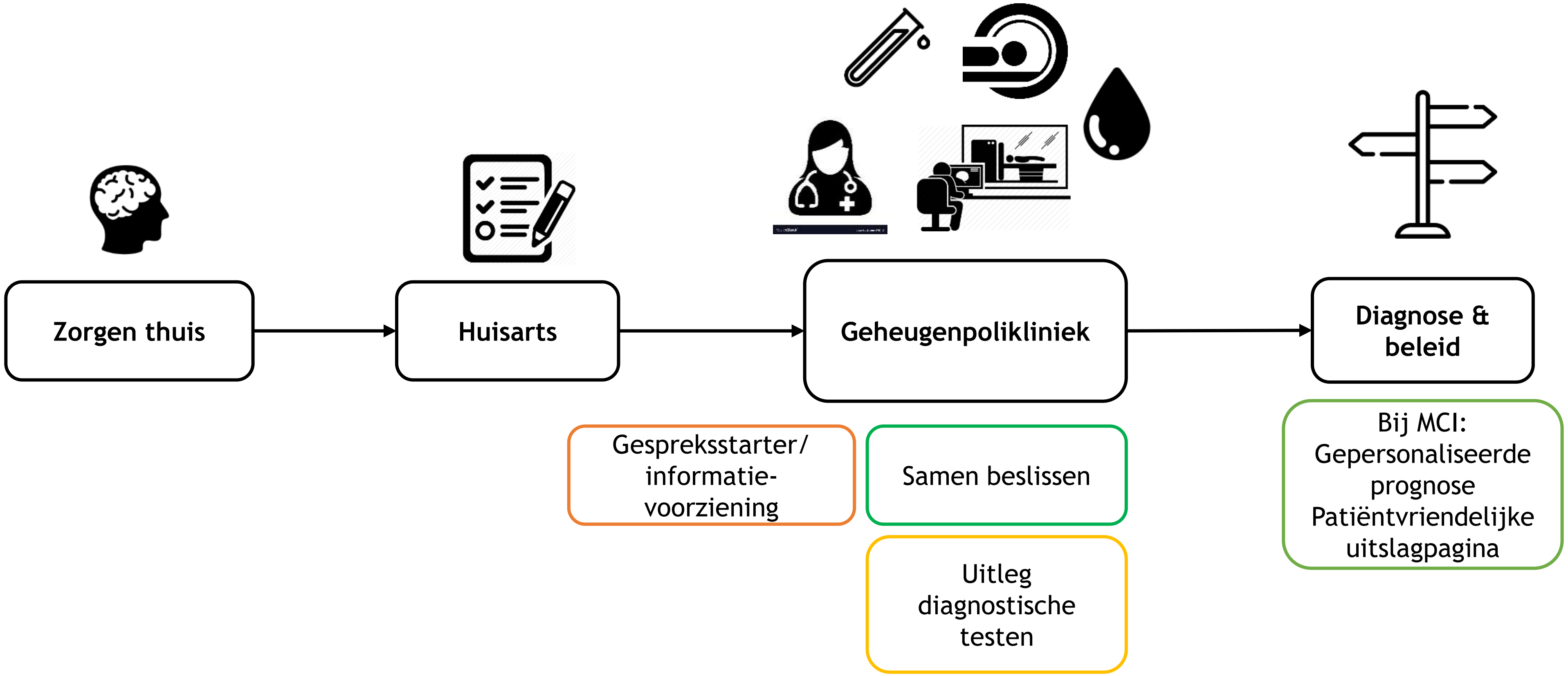

Casus mw. Van Dijk - geheugenpoli

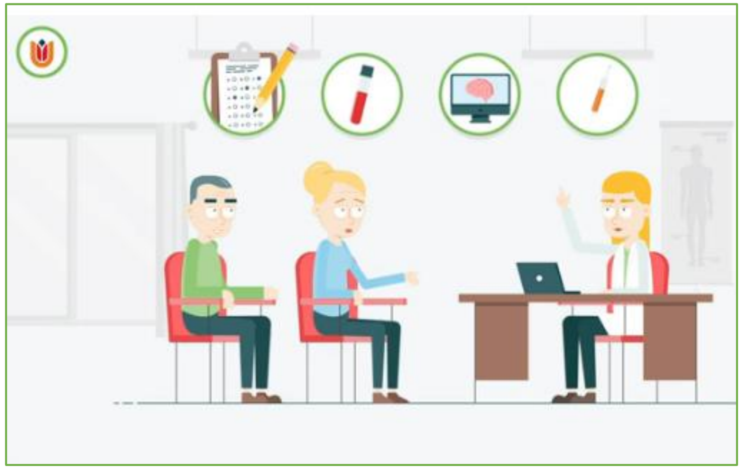

Voorafgaand aan de eerste afspraak

- Filmpje over wat te verwachten van de eerste afspraak

De eerste afspraak - Alzheimercentrum Amsterdam – YouTube

- Gespreksstarter: lijst met voorbeeldvragen

→ Welke onderwerpen moeten volgens zorgprofessionals, patiënten en naasten aan bod komen in het consult?

→Beter voorbereide en geïnformeerde patiënt

Casus mw. Van Dijk

Tijdens de eerste afspraak

- Vraag naar ingevulde lijst met voorbeeldvragen

- MMSE 26/30, MOCA 23/30, GDS 7/15, FAB 17/18, Benoemen 19/20
- DD: SCD/MCI obv AD of toch stemmingsproblematiek
- NPO? MRI? LP?

Alzheimercentrum Amsterdam
Amsterdam UMC

De gesprekswijzer: Welke vragen heeft u?

U heeft een afspraak in een geheugenpolikliniek. Deze gesprekswijzer met voorbeeldvragen kunt u printen en meenemen naar uw afspraak op de poli.

Kruis hieronder de vragen aan die u wilt stellen aan de arts. Heeft u andere vragen? Onderaan de pagina kunt u deze vragen opschrijven.

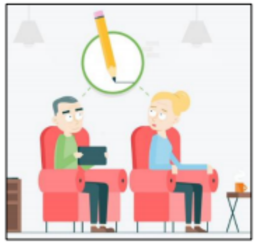

Dit wil ik vragen:

- [x] Wat kan ik verwachten dat er in de geheugenpolikliniek gaat gebeuren?
- [ ] Welke testen of onderzoeken zijn er mogelijk?
- [x] Wat zou er uit het onderzoek kunnen komen?
- [ ] Wat is de reden om een bepaald onderzoek wel of niet bij mij te doen?
- [ ] Wat is de uitslag van de onderzoeken die bij mij zijn gedaan? (Bijvoorbeeld een hersenscan, neuropsychologisch onderzoek, of ruggenprik)
- [ ] Wat zegt de uitslag van het onderzoek over de oorzaak van mijn klachten?
- [ ] Hoe zeker is deze uitslag?
- [ ] Wat heb ik precies en wat betekent dat?
- [ ] Heeft dit gevolgen voor mij? Bijvoorbeeld:
- [ ] Moet ik nu zorg regelen?
- [ ] Mag ik nog autorijden? Heeft dit gevolgen voor mijn rijbewijs?
- [ ] Moet ik medicijnen gaan gebruiken?
- [ ] Wat kan ik verwachten dat er gaat gebeuren in de toekomst?
- [ ] Wat kan ik zelf doen?
- [ ] Wie neemt mijn zorg na deze afspraak over?

Overige vragen:

Heb ik Alzheimer???

Casus mw. Van Dijk - geheugenpoli

Tijdens de <u>eerste afspraak</u>

- **Samen beslissen** over diagnostische testen d.m.v.:
  - Module ‘Gesprekswijzer Samen beslissen’

Voorbeeld: 'Over de verschillende testen valt veel te vertellen. Sommige patiënten willen daar graag zoveel mogelijk over horen, en anderen willen liever niet te veel weten. Hoe is dat voor u?'

Voorbeeld: 'Sommige mensen willen graag mee beslissen over diagnostisch testen, anderen laten dat liever aan de arts over. Hoe staat u daarin?'

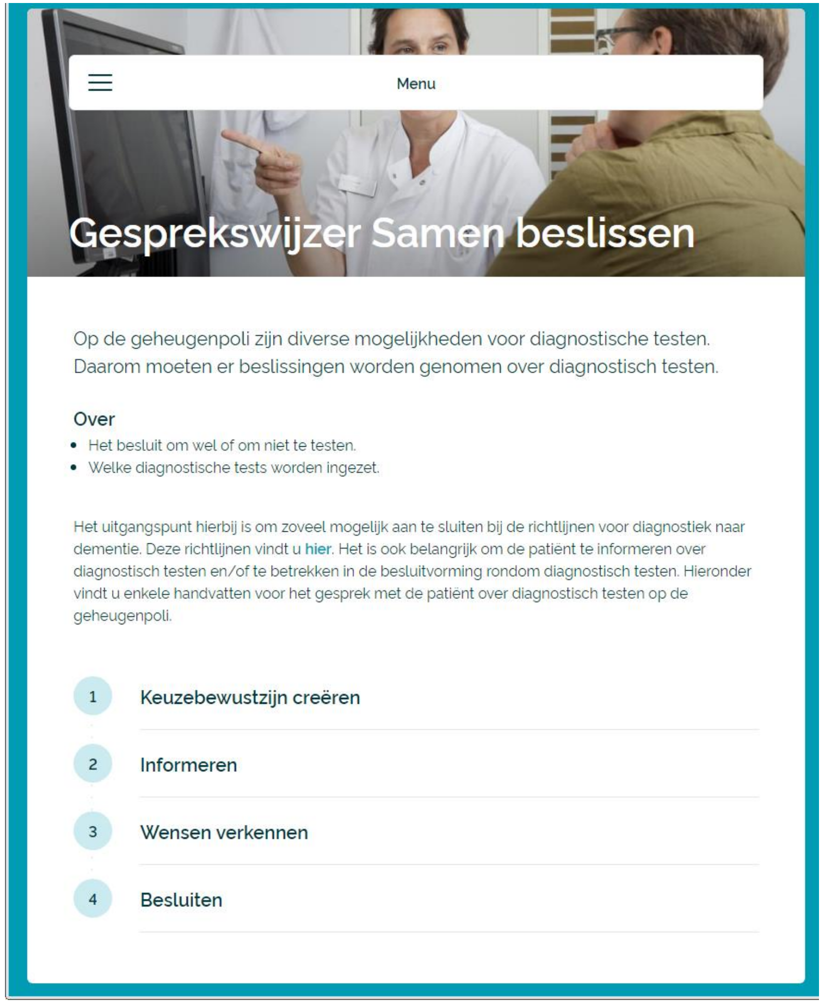

Casus mw. Van Dijk - geheugenpoli

Tijdens de eerste afspraak

- Samen beslissen over diagnostische testen d.m.v.:
  - Module ‘Gesprekswijzer Samen beslissen’
  - Module ‘Diagnostische testen’
    - Overzicht van veelvoorkomende testen met mogelijke voor- en nadelen

Neuropsychologisch onderzoek

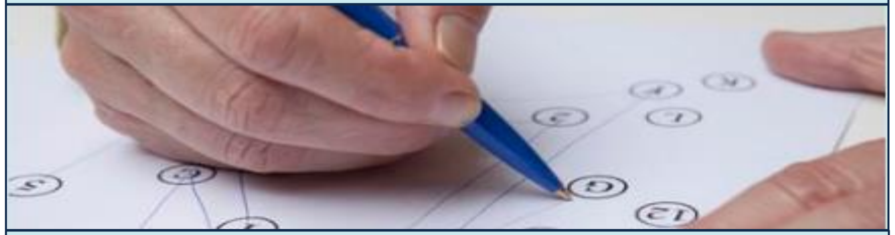

Voordelen:

- Hiermee weten we precies wat er mis gaat met het denken (‘cognitieve functies’). Bijvoorbeeld: Is de vergeetachtigheid erger dan verwacht voor iemand van uw leeftijd? U heeft geheugenklachten, komt dit door het geheugen of is het een concentratieprobleem?
- Het neuropsychologisch onderzoek meet of er sprake is van dementie. En het meet ook de ernst daarvan: milde, matige of ernstige dementie.

Nadelen:

- Sommige patiënten ervaren het neuropsychologisch onderzoek als confronterend, want ze merken wat er niet goed gaat.
- Omdat het testonderzoek vrij lang duurt, is het voor sommige mensen vermoeiend.

Casus mw. Van Dijk - geheugenpoli

Tijdens de <u>eerste afspraak</u>

- **Samen beslissen** over diagnostische testen d.m.v.:
  - Module ‘Gesprekswijzer Samen beslissen’
  - Module ‘Diagnostische testen’
    - Overzicht van veelvoorkomende testen met mogelijke voor- en nadelen

- **Alles besproken**? → Module ‘Topic lijst’ als checklist

→ Welke onderwerpen moeten volgens zorgprofessionals, patiënten en naasten aan bod komen in het consult?

(lijst met voorbeeldvragen is hierop gebaseerd)

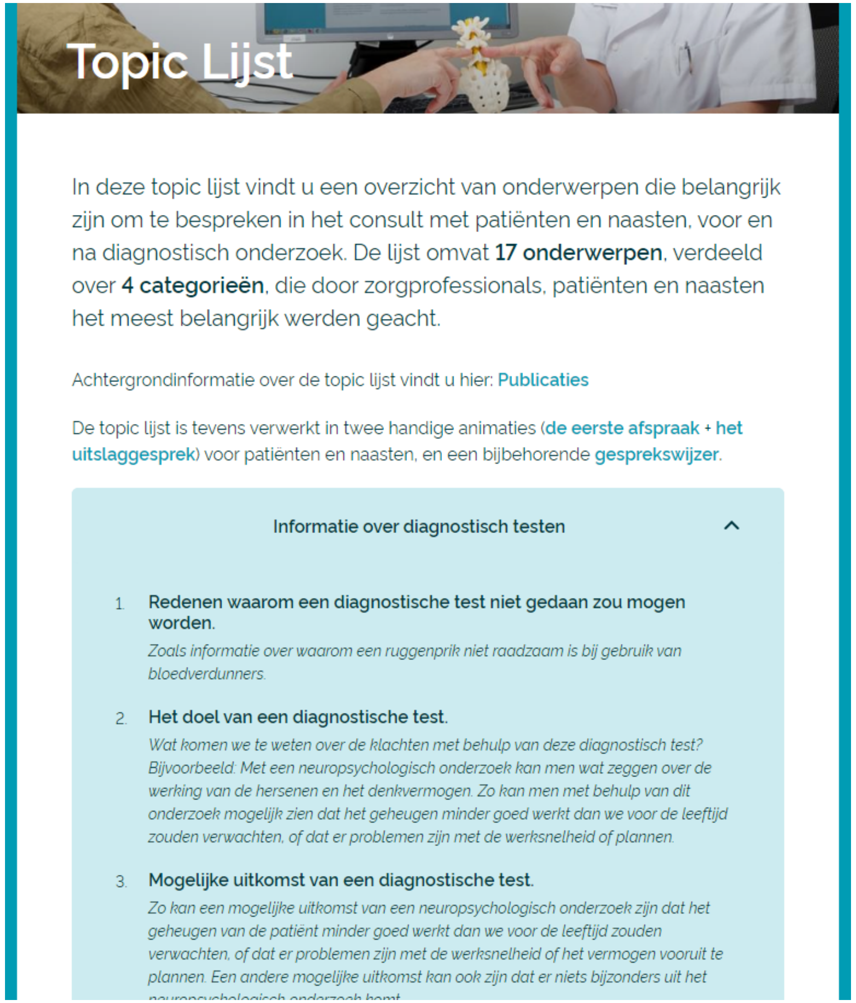
Topic Lijst

In deze topic lijst vindt u een overzicht van onderwerpen die belangrijk zijn om te bespreken in het consult met patiënten en naasten, voor en na diagnostisch onderzoek. De lijst omvat **17 onderwerpen**, verdeeld over **4 categorieën**, die door zorgprofessionals, patiënten en naasten het meest belangrijk werden geacht.

Achtergrondinformatie over de topic lijst vindt u hier: Publicaties

De topic lijst is tevens verwerkt in twee handige animaties (de eerste afspraak + het uitslaggesprek) voor patiënten en naasten, en een bijbehorende gesprekswijzer.

Informatie over diagnostisch testen

1. **Redenen waarom een diagnostische test niet gedaan zou mogen worden.**
   Zoals informatie over waarom een ruggenprik niet raadzaam is bij gebruik van bloedverdunners.
2. **Het doel van een diagnostische test.**
   Wat komen we te weten over de klachten met behulp van deze diagnostisch test? Bijvoorbeeld: Met een neuropsychologisch onderzoek kan men wat zeggen over de werking van de hersenen en het denkvermogen. Zo kan men met behulp van dit onderzoek mogelijk zien dat het geheugen minder goed werkt dan we voor de leeftijd zouden verwachten, of dat er problemen zijn met de werksnelheid of plannen.
3. **Mogelijke uitkomst van een diagnostische test.**
   Zo kan een mogelijke uitkomst van een neuropsychologisch onderzoek zijn dat het geheugen van de patiënt minder goed werkt dan we voor de leeftijd zouden verwachten, of dat er problemen zijn met de werksnelheid of het vermogen vooruit te plannen. Een andere mogelijke uitkomst kan ook zijn dat er niets bijzonders uit het [illegible]

Casus mw. Van Dijk - geheugenpoli

- NPO: stoornissen leervermogen, overige testresultaten binnen de norm
- MRI: MTA 2/2, GCA 1, PCA 2/2, Fazekas 1
- LP: Abeta 1-42 (Elecsys): 800*, Totaal Tau: 281*, pTau-181: 32*

Voorafgaand aan het uitslaggesprek

- Syndroomdiagnose MCI
- “50% kans op dementie”
- Gepersonaliseerde prognose met ADappt

www.ADappt.health → probeer het zelf!

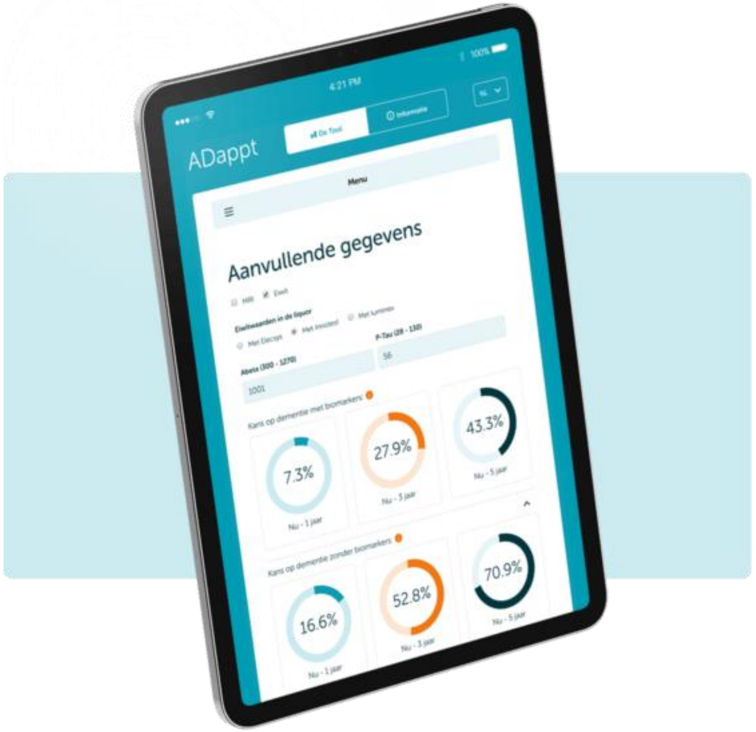

ADappt

Predictietool | Informatie | NL

Over de predictietool

Basisgegevens invoeren

Biomarkers toevoegen

Uitslagpagina patiënt

Over de predictietool

De ADappt predictietool geeft een schatting van de kans dat uw patiënt met milde cognitieve stoornissen (MCI) binnen nu en 1 tot 5 jaar dementie ontwikkelt.

Deze schatting wordt gemaakt op basis van leeftijd, geslacht en Mini Mental State Examination (MMSE) score (de kans zonder biomarkers).

Met aanvullende gegevens over de beoordeling van een MRI, amyloid beta 1-42 en gefosforyleerd tau waarden in liquor en/of amyloid PET kan een aangepaste schatting van de kans op dementie worden gegeven (de kans met biomarkers).

Let op: predictietool is alléén te gebruiken voor syndroomdiagnose MCI

Start met invoer basisgegevens

Privacy Statement

DISCLAIMER

VUmc kan geen enkele verantwoordelijkheid en/of aansprakelijkheid aanvaarden voor schade ten gevolge van...

Lees meer

ADappt

Predictietool | Informatie | NL

- **Over de predictietool**
- Basisgegevens invoeren
- Biomarkers toevoegen
- Uitslagpagina patiënt

Over de predictietool

De ADappt predictietool geeft een schatting van de kans dat uw patiënt met milde cognitieve stoornissen (MCI) binnen nu en 1 tot 5 jaar dementie ontwikkelt.

Deze schatting wordt gemaakt op basis van leeftijd, geslacht en Mini Mental State Examination (MMSE) score (de kans zonder biomarkers).

Met aanvullende gegevens over de beoordeling van een MRI, amyloid beta 1-42 en gefosforyleerd tau waarden in liquor en/of amyloid PET kan een aangepaste schatting van de kans op dementie worden gegeven (de kans met biomarkers).

Start met invoer basisgegevens

Privacy Statement

DISCLAIMER
VUmc kan geen enkele verantwoordelijkheid en/of aansprakelijkheid aanvaarden voor schade ten gevolge van...
Lees meer

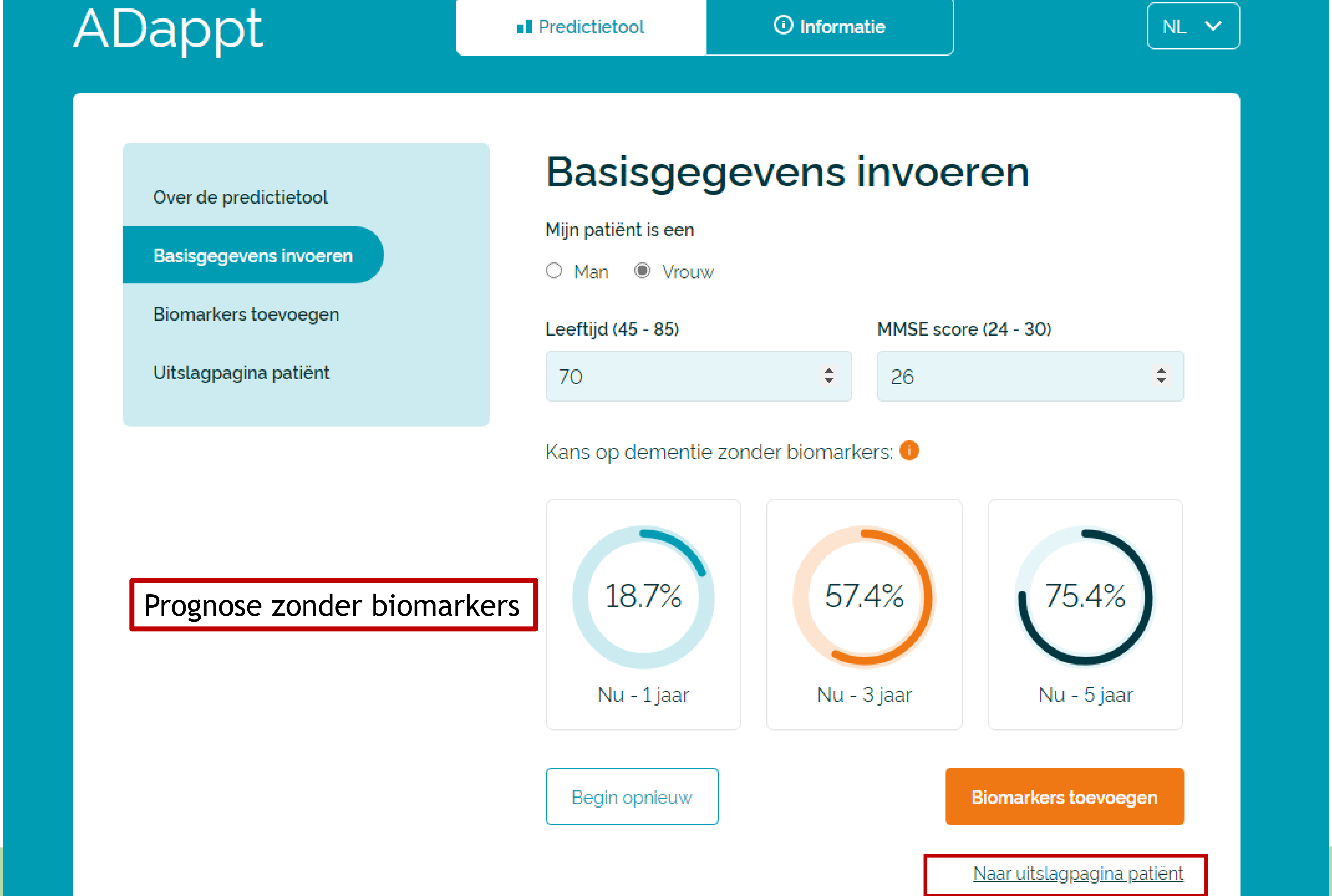
ADappt
Predictietool
Informatie
NL
Over de predictietool
Basisgegevens invoeren
Biomarkers toevoegen
Uitslagpagina patiënt
Basisgegevens invoeren
Mijn patiënt is een
Man
Vrouw
Leeftijd (45 - 85)
70
MMSE score (24 - 30)
26
Kans op dementie zonder biomarkers:
18.7%
Nu - 1 jaar
57.4%
Nu - 3 jaar
75.4%
Nu - 5 jaar
Prognose zonder biomarkers
Begin opnieuw
Biomarkers toevoegen
Naar uitslagpagina patiënt

ADappt

Predictietool | Informatie | NL

Over de predictietool

Basisgegevens invoeren

Biomarkers toevoegen

Uitslagpagina patiënt

Basisgegevens invoeren

Mijn patiënt is een

○ Man ◉ Vrouw

Leeftijd (45 - 85)

70

MMSE score (24 - 30)

26

Kans op dementie zonder biomarkers:

18.7%	57.4%	75.4%
Nu - 1 jaar	Nu - 3 jaar	Nu - 5 jaar

Begin opnieuw

Biomarkers toevoegen

Naar uitslagpagina patiënt

Over de predictietool

Basisgegevens invoeren

Biomarkers toevoegen

Uitslagpagina patiënt

Biomarkers toevoegen

Welke biomarkers wilt u toevoegen?

- [x] MRI
- [x] Eiwitwaarden
- [ ] Amyloid-PET

Beoordeling MRI

(•) Visueel ( ) Volumetrisch

MTA links (0 - 4)	MTA rechts (0 - 4)
2	2

Eiwitwaarden in de liquor

(•) Met Elecsys ( ) Met Innotest ( ) Met Luminex

Abeta (390 - 1900)	P-Tau (10 - 50)
800	32

Kans op dementie met biomarkers:

6.3%	62.4%	90.2%
Nu - 1 jaar	Nu - 3 jaar	Nu - 5 jaar

Kans op dementie zonder biomarkers:

Begin opnieuw

Naar uitslagpagina patiënt

Let op: deze informatie is voor de zorgverlener, voor de patiënt is een patiëntvriendelijke uitslagpagina ontwikkeld

Uitslagpagina patiënt

Uw situatie

Bij u is sprake van milde cognitieve klachten. Deze diagnose 'MCI' betekent ook dat u een verhoogde kans heeft om in de toekomst dementie te ontwikkelen.

Bij u zijn aanvullende diagnostische tests uitgevoerd. Deze tests tonen aan of er in uw hersenen al sprake is van Alzheimer-schade. Het is mogelijk om Alzheimer-schade te hebben, zonder dat er sprake is van dementie. Maar deze schade geeft wel een verhoogde kans op dementie in de komende jaren.

MRI

Een MRI is een foto van uw hersenen. Hier ziet u een voorbeeld van een MRI van gezonde hersenen. Op een MRI kunnen we zien of er sprake is van hersenkrimp (atrofie).

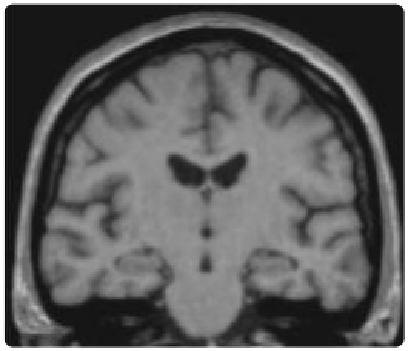

In uw geval is er sprake van hersenkrimp. Hierdoor heeft u een iets hogere kans om de komende jaren dementie te ontwikkelen.

Duidelijk geen krimp | Niet duidelijk of er krimp is | **Duidelijk wel krimp (atrofie)**

Met de ruggenprik (lumbaalpunctie) tonen we aan of er aanwijzingen zijn voor de aanwezigheid van de Alzheimer-eiwitten in de hersenen.

Er zijn 2 soorten eiwitten: amyloid en p-tau.

800
Amyloid

32
Tau

Hieruit maken we op dat er Alzheimer-eiwitten aanwezig zijn in de hersenen. Dit betekent dat u een hogere kans heeft om de komende jaren dementie te ontwikkelen.

Duidelijk geen Alzheimer-eiwitten aanwezig | Niet duidelijk of er Alzheimer-eiwitten aanwezig zijn | **Duidelijk wel Alzheimer-eiwitten aanwezig**

Wat betekent dit voor u de komende tijd?

Om een uitspraak te kunnen doen over de kans dat uw klachten zich ontwikkelen tot dementie, vergelijken we uw situatie met andere vrouwelijke patiënten in onze kliniek, met dezelfde leeftijd en resultaten op diagnostische tests als u.

Van die vrouwen in dezelfde situatie als u, ontwikkelen er 6 van de 100 binnen nu en 1 jaar dementie.

Als we een langere periode bekijken, dan ontwikkelen 62 van de 100 vrouwen in dezelfde situatie als u binnen nu en 3 jaar dementie.

Op nog langere termijn, dan ontwikkelen 90 van de 100 vrouwen in dezelfde situatie als u binnen nu en 5 jaar dementie.

Hieronder ziet u een visuele weergave van de kans op het ontwikkelen van dementie voor 100 vrouwen van uw leeftijd, met dezelfde resultaten op de diagnostische tests, binnen nu en 1 jaar, 3 jaar en 5 jaar.

1 jaar

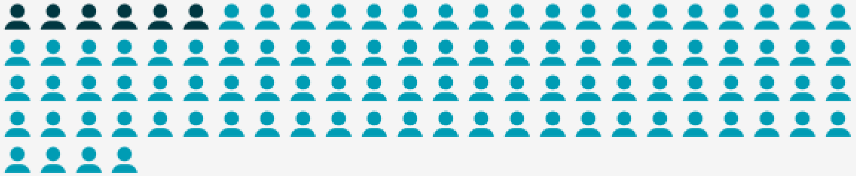

6 mensen ontwikkelen binnen nu en 1 jaar dementie.

94 mensen ontwikkelen binnen nu en 1 jaar geen dementie.

3 jaar

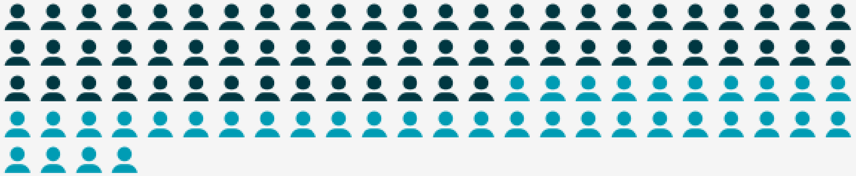

62 mensen ontwikkelen binnen nu en 3 jaar dementie.

38 mensen ontwikkelen binnen nu en 3 jaar geen dementie.

5 jaar

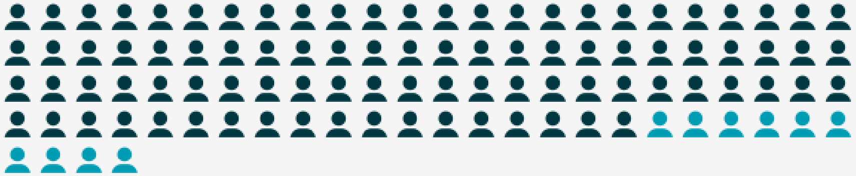

90 mensen ontwikkelen binnen nu en 5 jaar dementie.

10 mensen ontwikkelen binnen nu en 5 jaar geen dementie.

Wat betekent dit voor u de komende tijd?

Om een uitspraak te kunnen doen over de kans dat uw klachten zich ontwikkelen tot dementie, vergelijken we uw situatie met andere vrouwelijke patiënten in onze kliniek, met dezelfde leeftijd en resultaten op diagnostische tests als u.

Van die vrouwen in dezelfde situatie als u, ontwikkelen er 6 van de 100 binnen nu en 1 jaar dementie.

Als we een langere periode bekijken, dan ontwikkelen 62 van de 100 vrouwen in dezelfde situatie als u binnen nu en 3 jaar dementie.

Op nog langere termijn, dan ontwikkelen 90 van de 100 vrouwen in dezelfde situatie als u binnen nu en 5 jaar dementie.

Aanvullen of aanpassen

- ADappt slaat ivm privacy geen gegevens op → meteen printen of tijdens consult opnieuw doen
- Bespreek of patiënt gepersonaliseerde prognose wil!

Casus mw. Van Dijk - geheugenpoli

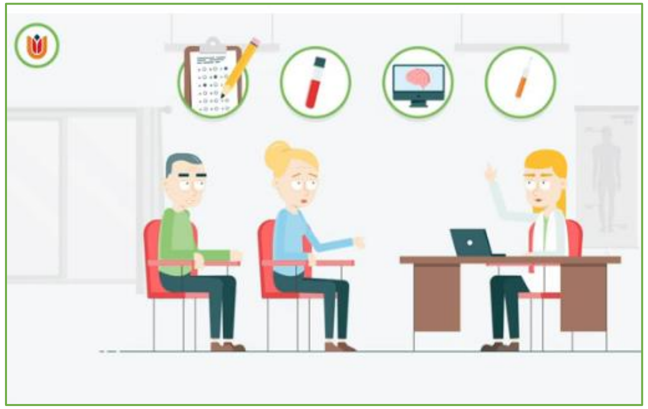

Voorafgaand aan het <u>uitslaggesprek</u>

- Filmpje over wat te verwachten van het uitslaggesprek

[Het uitslaggesprek - Alzheimercentrum Amsterdam – YouTube]

- Gespreksstarter: lijst met voorbeeldvragen

→ Welke onderwerpen moeten volgens zorgprofessionals, patiënten en naasten aan bod komen in het consult?

Casus mw. Van Dijk

Tijdens het <u>uitslaggesprek</u>

- Bespreek resultaten aan de hand van uitslagpagina
- Geef een print mee
- Aanvullende diagnostiek? → Samen beslissen
- Vraag naar ingevulde lijst met voorbeeldvragen
- Alles besproken? Check module 'Topic lijst'

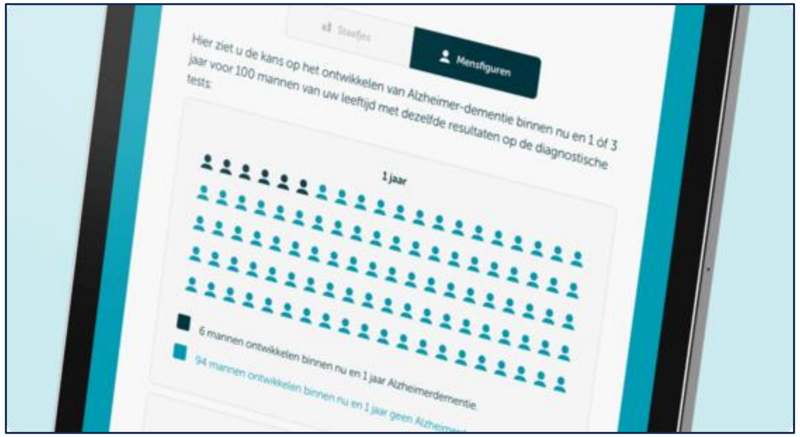

Alzheimercentrum Amsterdam
Amsterdam UMC

De gesprekswijzer: Welke vragen heeft u?

U heeft een afspraak in een geheugenpolikliniek. Deze gesprekswijzer met voorbeeldvragen kunt u printen en meenemen naar uw afspraak op de poli.

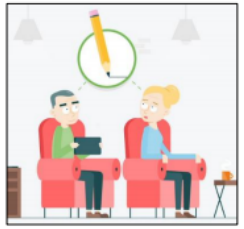

Kruis hieronder de vragen aan die u wilt stellen aan de arts. Heeft u andere vragen? Onderaan de pagina kunt u deze vragen opschrijven.

Dit wil ik vragen:

- [ ] Wat kan ik verwachten dat er in de geheugenpolikliniek gaat gebeuren?
- [ ] Welke testen of onderzoeken zijn er mogelijk?
- [ ] Wat zou er uit het onderzoek kunnen komen?
- [ ] Wat is de reden om een bepaald onderzoek wel of niet bij mij te doen?
- [ ] Wat is de uitslag van de onderzoeken die bij mij zijn gedaan? (Bijvoorbeeld een hersenscan, neuropsychologisch onderzoek, of ruggenprik)
- [x] Wat zegt de uitslag van het onderzoek over de oorzaak van mijn klachten?
- [ ] Hoe zeker is deze uitslag?
- [ ] Wat heb ik precies en wat betekent dat?
- [ ] Heeft dit gevolgen voor mij? Bijvoorbeeld:
- [x] <u>Moet ik nu zorg regelen</u>?
- [ ] Mag ik nog autorijden? Heeft dit gevolgen voor mijn rijbewijs?
- [ ] Moet ik medicijnen gaan gebruiken?
- [ ] Wat kan ik verwachten dat er gaat gebeuren in de toekomst?
- [ ] Wat kan ik zelf doen?
- [ ] Wie neemt mijn zorg na deze afspraak over?

Overige vragen:

Heb ik Alzheimer???

ADappt – samengevat:

- **Beter voorbereide en geïnformeerde** patiënten en naasten
- **Samen beslissen** over diagnostische testen

Voor patiënten met de diagnose 'mild cognitive impairment' (MCI)

- Ondersteuning voor de arts bij het **interpreteren van de (biomarker) test uitslagen** van patiënten met MCI
- **Meer zekerheid** over de uitslag en een **uitslagpagina** voor patiënten met MCI

ADappt - samengevat:

ADappt kan op **3** verschillende momenten worden gebruikt:

- Ter voorbereiding op / tijdens **het eerste consult**
- **Tijdens het MDO** (alleen zorgverlener)
- Ter voorbereiding op / tijdens **het uitslaggesprek**

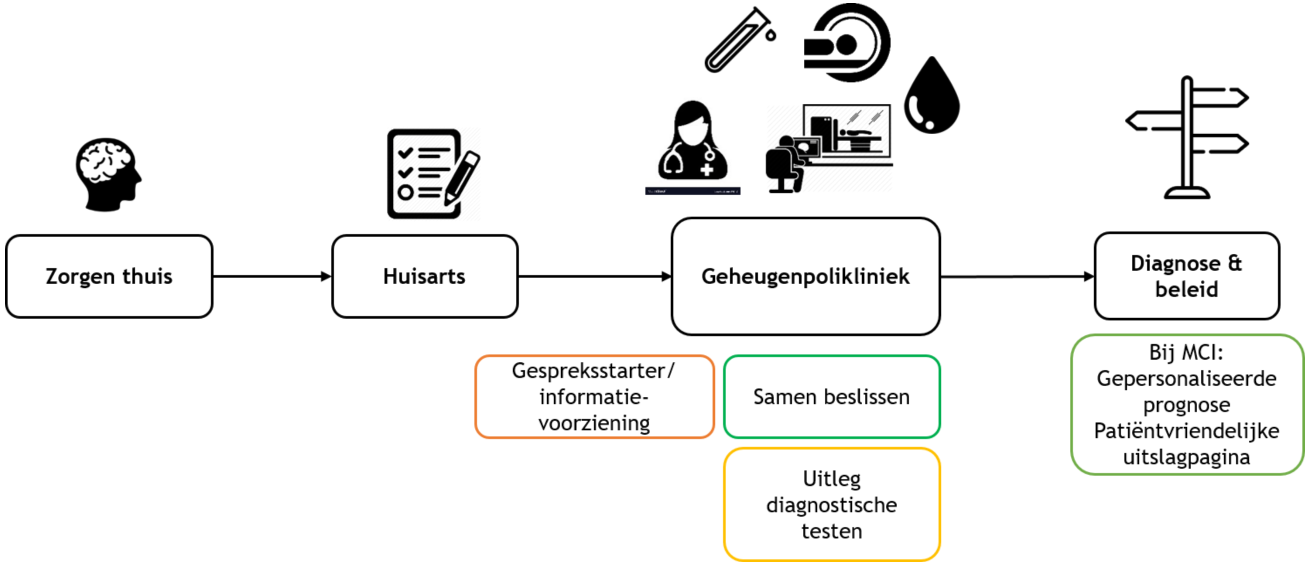

ADappt – samengevat:

Dit is hoe wij het gebruik van ADappt voor ons zien

- Maar: experimenteer
- Kijk wat voor u werkt/niet werkt
- Wat u wel of niet bruikbaar vindt

→input voor haalbaarheidsstudie

Haalbaarheidsstudie

- Evaluaties, ervaringen en ideeën voor ADappt

- Wat vinden zorgverleners, patiënten en naasten van ADappt?
- Lukt het om ADappt in de praktijk te gebruiken? Waarom wel/niet?
- Op welke manier wordt ADappt gebruikt?
- Welke modules worden gebruikt?

Hoe?

- Patiënten en naasten: vragenlijsten, audio-opname
- Clinici: vragenlijsten, één telefonisch interview, audio-opname

Participant journey

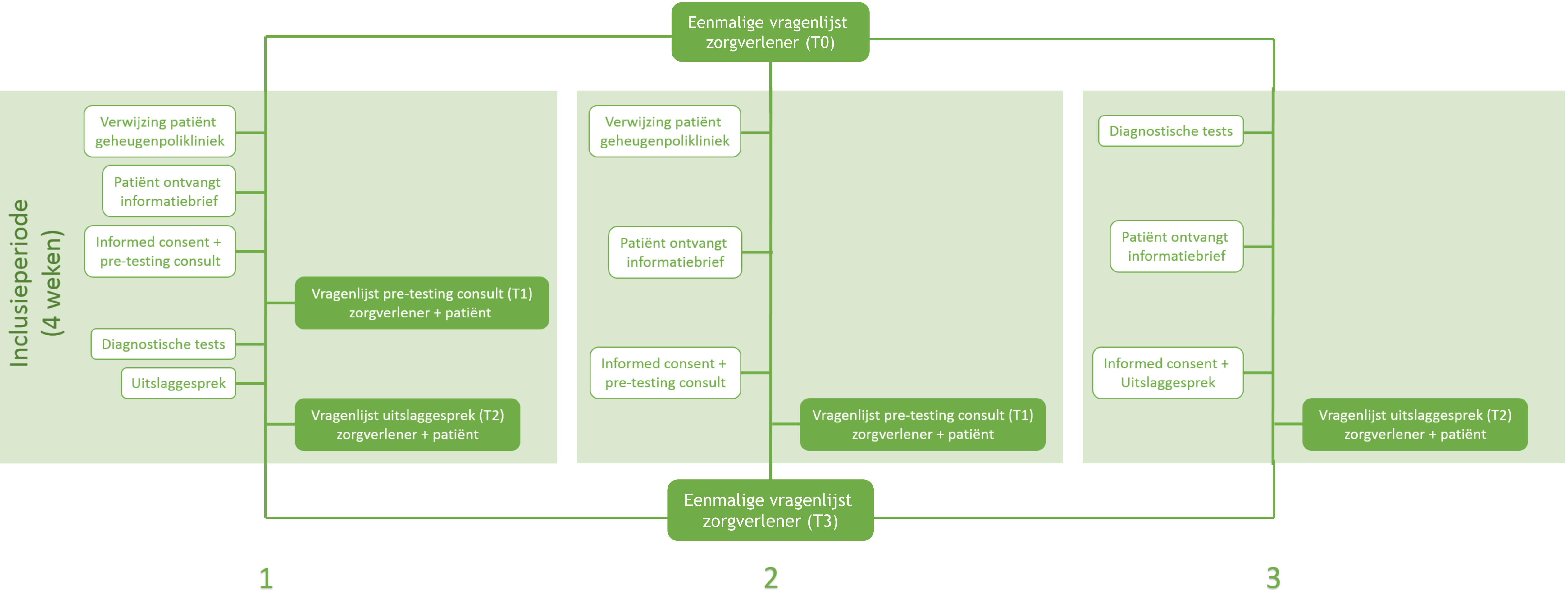

Haalbaarheidsstudie

- Wat is er nodig om de haalbaarheidsstudie te starten?
- Hoe kunnen we jullie daarin zoveel mogelijk ondersteunen?

- Vragen?

Contact:

[*e-mailadres*]
