# Supplementary material for: Usability and feasibility of ADappt: a digital toolkit to support communication on diagnosis and prognosis in memory clinics
Source: Alzheimers Res Ther. 2025 Oct 2;17:218. doi: 10.1186/s13195-025-01847-y (PMC12492680; doi:10.1186/s13195-025-01847-y)
Supplement: Supplementary file 8 — Supplementary Material 8 [file 13195_2025_1847_MOESM8_ESM.pdf]

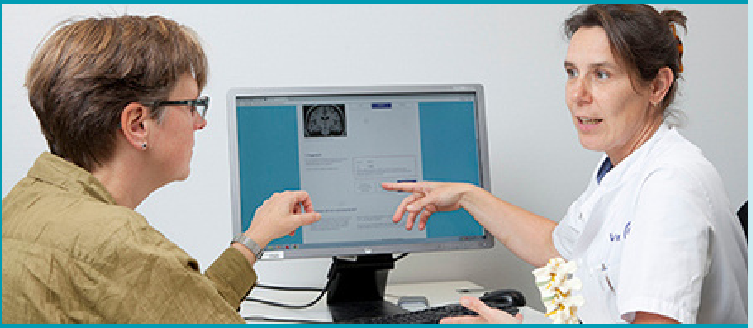

Contactgegevens

●●●

[naam]
[functie]
[organisatie]
[adres]
[e-mailadres]
[telefoonnummer]

ADappt

Gebruiksaanwijzing voor patiënten en naasten

Wat is ADappt?

Op www.ADappt.health staan hulpmiddelen om u voor te bereiden op uw afspraken in de geheugenpolikliniek.

- Korte informatievideo's over de eerste afspraak en over het uitslaggesprek op de geheugenpoli.
- Een gesprekswijzer: een lijst met voorbeeldvragen die u kunt invullen en meenemen naar zowel de eerste afspraak als het uitslaggesprek.

Heb ik het niet duidelijk uitgelegd? Op de achterzijde vindt u mijn contactgegevens. Neem gerust contact met mij op!

Wat is het nut van ADappt?

- Door de informatievideo's te bekijken, heeft u een idee van wat u kunt verwachten tijdens de afspraak.

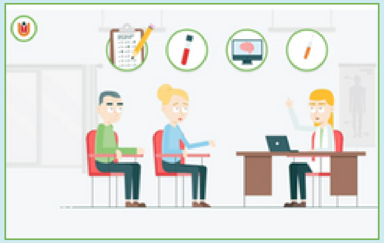

- De gesprekswijzer kunt u meenemen naar de afspraak. Zo is duidelijk welke vragen u wilt stellen. Dit kan uzelf en de zorgverlener helpen tijdens het gesprek.

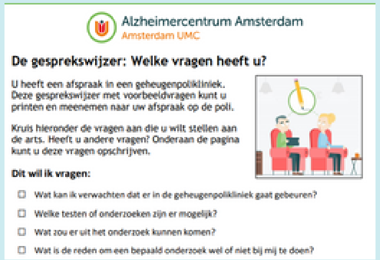

Alzheimercentrum Amsterdam
Amsterdam UMC

De gesprekswijzer: Welke vragen heeft u?

U heeft een afspraak in een geheugenpolikliniek. Deze gesprekswijzer met voorbeeldvragen kunt u printen en meenemen naar uw afspraak op de poli.

Kruis hieronder de vragen aan die u wilt stellen aan de arts. Heeft u andere vragen? Onderaan de pagina kunt u deze vragen opschrijven.

Dit wil ik vragen:

- ☐ Wat kan ik verwachten dat er in de geheugenpolikliniek gaat gebeuren?
- ☐ Welke testen of onderzoeken zijn er mogelijk?
- ☐ Wat zou er uit het onderzoek kunnen komen?
- ☐ Wat is de reden om een bepaald onderzoek wel of niet bij mij te doen?

Waar vindt u de hulpmiddelen?

01. Ga naar www.adappt.health

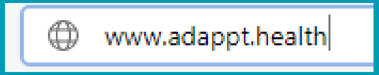

02. Aan de linkerkant van de pagina vindt u het menu. Klik in het menu op het tabblad "Topic lijst"

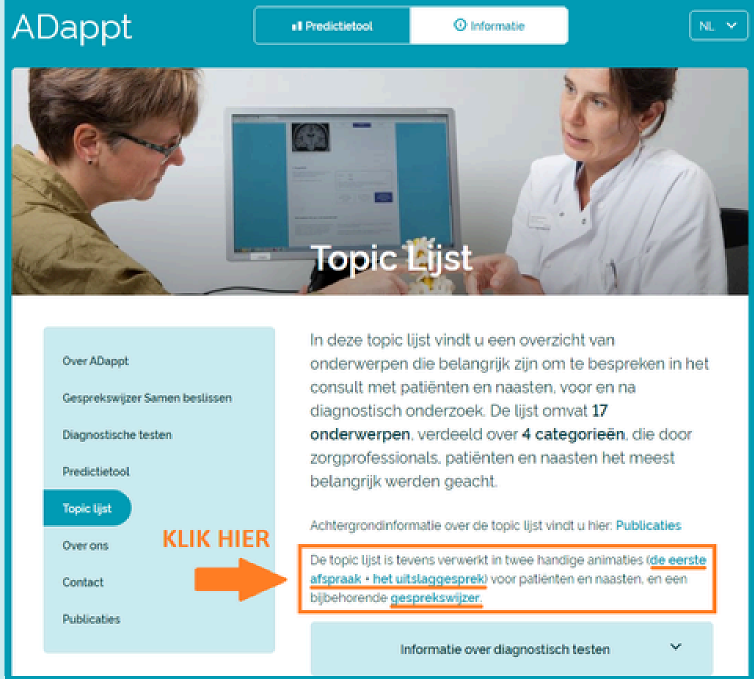

03. Voor de informatievideo's, klik op "de eerste afspraak" of "het uitslaggesprek"

04. Voor de gesprekswijzer, klik op "gesprekswijzer"
